# Cognitive behavioral therapy for eating disorders: A map of the systematic review evidence base

**DOI:** 10.1002/eat.23831

**Published:** 2022-10-31

**Authors:** Milla Kaidesoja, Zafra Cooper, Beth Fordham

**Affiliations:** ^1^ NDORMS University of Oxford Oxford UK; ^2^ Department of Psychiatry Yale School of Medicine New Haven Connecticut USA

**Keywords:** anorexia nervosa, binge‐eating disorder, bulimia nervosa, CBT, cognitive behavioral therapy, eating disorder, EDNOS, OSFED, overview, systematic review

## Abstract

**Objective:**

To map and examine the systematic review evidence base regarding the effects of cognitive‐behavioral therapy (CBT) for eating disorders (EDs), especially against active interventions.

**Method:**

This systematic review is an extension of an overview of CBT for all health conditions (CBT‐O). We identified ED‐related systematic reviews from the CBT‐O database and performed updated searches of EMBASE, MEDLINE, and PsychInfo in April 2021 and September 2022.

**Results:**

The 44 systematic reviews included (21 meta‐analyses) were of varying quality. They focused on “high intensity” CBT, delivered face‐to‐face by qualified clinicians, in BN, BED and mixed, not specifically low‐weight samples. ED‐specific outcomes were studied most, with little consensus on their operationalization. The, often insufficient, reporting of sample characteristics did not allow assessment of the generalizability of findings. The meta‐analytic syntheses show that high intensity one‐to‐one CBT produces better short‐term effects than a mix of active controls especially on ED‐specific measures for BED, BN, and transdiagnostic samples. There is little evidence favoring group CBT or low intensity CBT against other active interventions.

**Discussion:**

While this study found evidence consistent with current ED treatment recommendations, it highlighted notable gaps that need to be addressed. There were insufficient data to allow generalizations regarding sex and gender, age, culture and comorbidity and to support CBT in AN samples. The evidence for group CBT and low intensity CBT against active controls is limited, as it is for the longer‐term effects of CBT. Our findings identify areas for future innovation and research within CBT.

**Public Significance:**

This study provides a comprehensive mapping and quality assessment of the current large systematic review research base regarding the effects of cognitive behavioral therapy (CBT) for eating disorders (EDs), with a focus on comparisons to other active interventions. By transcending the more limited scope of individual systematic reviews, this overview highlights the gaps in the current evidence base, and thus provides guidance for future research and clinical innovation.

## Resumen

**Objetivo:**

Mapear y examinar la base de evidencia de la revisión sistemática con respecto a los efectos de la terapia cognitivo‐conductual (TCC) para los trastornos de la conducta alimentaria (TCA), especialmente contra las intervenciones activas.

**Método:**

Esta revisión sistemática es una extensión de una visión general de la TCC para todas las afecciones de salud (TCC‐O, Fordham et al., 2021a). Se identificaron revisiones sistemáticas relacionadas con los TCA a partir de la base de datos TCC‐O y se realizaron búsquedas actualizadas en EMBASE, MEDLINE y PsychInfo en abril de 2021 y septiembre de 2022.

**Resultados:**

Las 44 revisiones sistemáticas incluidas (21 metanálisis) fueron de calidad variable. Se centraron en la TCC de “alta intensidad”, administrada cara a cara por clínicos calificados, en BN, TpA y muestras mixtas, no específicamente de bajo peso. Los resultados específicos de los TCA fueron los más estudiados, con poco consenso sobre su operacionalización. El informe, a menudo insuficiente, de las características de la muestra no permitió evaluar la generalización de los hallazgos. Las síntesis metaanalíticas muestran que la TCC uno a uno de alta intensidad produce mejores efectos a corto plazo que una combinación de controles activos, especialmente en medidas específicas de TCA para TpA, BN y muestras transdiagnósticas. Hay poca evidencia a favor de la TCC grupal o la TCC de baja intensidad frente a otras intervenciones activas.

**Discusión:**

Si bien este estudio encontró evidencia consistente con las recomendaciones actuales de tratamiento de los TCA, también destacó las brechas notables que deben abordarse. No hubo datos suficientes para permitir generalizaciones con respecto al sexo y el género, la edad, la cultura y la comorbilidad y para apoyar la TCC en las muestras de AN. La evidencia para la TCC grupal y la TCC de baja intensidad contra los controles activos es limitada, al igual que para los efectos a más largo plazo de la TCC. Nuestros hallazgos identifican áreas para la innovación y la investigación futuras dentro de la TCC.

## 1 INTRODUCTION

Cognitive behavioral approaches to the understanding and treatment of the eating disorders (EDs) were first developed in the early eighties (Fairburn, 1981; Fairburn et al., 1986; Garner & Bemis, 1982). Since this time, theory and treatment have evolved to focus on the mechanisms proposed to maintain eating disorder psychopathology across the full range of EDs (Cooper & Fairburn, 2011; Fairburn et al., 2003). In addition, evidence supporting cognitive behavioral therapy (CBT) for these disorders has accumulated from randomized controlled trials (RCTs) and has been synthesized in a number of systematic reviews (e.g., Bulik, Berkman, Brownley, Sedway, & Lohr, 2007; Hay, 2013; Linardon et al., 2017e). Further support has come from the use of evidence supported CBT in real world settings (Weissman et al., 2017).

CBT is now recommended by the majority of evidence‐based national guidelines (Hilbert et al., 2017) as the first line of treatment for bulimia nervosa (BN) and binge eating disorder (BED), and to a lesser extent (due to less robust evidence) for the other specified feeding and eating disorders (OSFED) (this DSM‐5 diagnosis partially overlaps with the previously used DSM IV category, “eating disorder not otherwise specified” [EDNOS]). While CBT is clearly regarded as the treatment of choice for these latter disorders that do not involve significantly low weight (Weissman et al., 2017), it is one amongst a number of options for the treatment of adults with anorexia nervosa (AN) (Mulkens & Waller, 2021). Three main approaches are currently recommended for the treatment of AN (e.g., National Institute for Health and Care Excellence [NICE], 2017) specialist supportive clinical management (SSCM; Carter et al., 2011; McIntosh et al., 2006; Touyz et al., 2013); the Maudsley model AN treatment for adults (MANTRA; Schmidt et al., 2015) and CBT (CBT‐E; Fairburn et al., 2013).

In sum, there is evidence from RCTs investigating the effectiveness of CBT in comparison to both active (other treatment interventions) and inactive control conditions in the various distinct eating disorder presentations as well as those investigating various delivery methods for CBT (e.g., group, individual, guided self‐help). Individual systematic reviews including meta‐analyses (MA) have been conducted for different treatment intervention types and different delivery modes for each of the distinct eating disorder presentations. To our knowledge there has been no comprehensive critical synthesis or overview of this large systematic review literature to map the extent and strength of the available evidence and to identify gaps in the systematic review evidence. Recently, an overview of CBT systematic reviews across all health conditions (CBT‐O) was published (Fordham et al., 2021a) that identified the large systematic review evidence base for EDs. However, due to the heterogeneity of the clinical presentations and outcomes, the overview did not focus specifically on reporting the evidence for CBT for EDs. The present study addressed this omission by conducting a continuation of the CBT‐O focusing exclusively on EDs. It aimed to provide a critical synthesis of this systematic review evidence to identify the extent and strength of the evidence for CBT, to identify gaps in the evidence drawn from individual systematic reviews and examine the quality of the reviews from which it is drawn. Critically synthesizing and evaluating the evidence at this higher level of generalization allows a meta‐perspective of all the systematic review evidence free of the more limited scope of any individual systematic review. An overview of systematic reviews also highlights the focus of the majority of past research in the area.

The present synthesis of evidence from systematic reviews regarding the effects of CBT for EDs aimed to examine:

1. the comparative effects of CBT in relation to other active treatments
2. the effects of different forms and delivery of CBT
3. whether CBT is transdiagnostic, achieving similar effects on the full range of eating disorder presentations
4. the populations (in terms of age, sex and gender, ethnic/cultural contexts and those with comorbid health conditions) for whom there are data regarding the effects of CBT
5. the longer‐term effects of CBT
6. the range of outcomes reported for CBT

## 2 METHODS

### 2.1 Search strategy

The present study is an extension of an overview of CBT for all health conditions (CBT‐O), (Fordham et al., 2021a; Fordham et al., 2021b). The full methods of the CBT‐O study have been previously published (Fordham, Suganvam, et al., 2021a). Where applicable, we adhered to the CBT‐O protocol and did not produce a separate protocol for this ED focused study. One author (MK) identified systematic reviews with ED related outcomes as part of the original search conducted for the CBT‐O study, and we subsequently conducted two updated searches, in April 2021 and September 2022, of EMBASE, MEDLINE and PsychInfo using the same search strategy as the original with these two additional search queries: (1) restricted to EDs and (2) publication dates of January 2019 to April 2021 and April 2021 to September 2022 respectively. Only systematic reviews were included since they are widely considered the gold standard method for evidence synthesis. Only papers written in English were included due to the authors' limited proficiency in other languages. A list of excluded papers with reasons for their exclusion (S1) and the details of the search strategy (S4) are provided in the Supplementary material.

### 2.2 Inclusion criteria

Inclusion criteria were those used in the CBT‐O study with minor modifications in line with our research questions. The criteria were as follows:

1. As in the CBT‐O study, reviews fulfilled at least four of the five criteria outlined by the widely accepted Centre for Reviews and Dissemination (CRD), as part of the Database of Abstracts of Reviews of Effects (DARE). These are: reporting of inclusion/exclusion criteria; adequacy of search; synthesis of included studies; assessment of quality of included studies and presentation of sufficient details about the individual studies (Khan et al., 2001).
2. Interventions studied were CBT treatments (excluding CBT in combination with other treatments, and prevention interventions). “Third‐wave” therapies (e.g., dialectical behavior therapy) were not included as CBT interventions.
3. CBT treatments were compared to non‐CBT control conditions.
4. Participants studied met full or subthreshold criteria for an ED (excluding the newly recognized feeding disorders).
5. Outcomes of the RCTs included in the reviews were qualitatively or quantitatively summarized.
6. Reviews were in English.

### 2.3 Data extraction

We based our data extraction template on a pre‐designed set of data tables from the CBT‐O study and amended it to better answer our research questions (see Supplementary material S3 for the amendments). The following data were extracted by one author (MK): demographic details (age group, sex and gender, ethnicity, comorbidity, diagnostic status [as reported in the systematic review], country), intervention details (type of CBT [high intensity/low intensity; ED‐specific or generic; individual or group, CBT protocol]), comparison details (active/inactive control, description of control), length of follow‐up (short = <12 months/long = ≥12 months), possible harms related to treatment, and data from MAs when provided (number of RCTs, type of comparison, outcomes studied, results and whether poor quality RCTs were excluded from the review). If a MA provided a synthesis at both end‐of‐treatment (EOT) and at short term follow‐up, in interests of parsimony only the EOT time point analysis was extracted since this was reported more often than the short follow‐up timepoint. CBT delivery was categorized as high or low intensity, based on Roth and Pilling (2007) with high‐intensity CBT defined as face‐to‐face, individual or group therapy, delivered by a trained CBT therapist and low‐intensity as delivered via media (internet, written, telephone), or face‐to‐face, individual or group CBT interventions delivered by a non‐CBT therapist (paraprofessional or layperson). Self‐help (including guided self‐help) was categorized as a low intensity intervention. If the review did not report the intensity of the intervention, it was assumed to be high intensity CBT. Also, if length of follow‐up was not clearly reported, it was assumed to be short.

### 2.4 Quality assessment

As in the CBT‐O study, the quality of the systematic reviews was assessed using the widely accepted AMSTAR‐2 (Shea et al., 2017). The individual item descriptions are provided in Table 1 in the Results. Reviews included in the original study had been previously assessed by the CBT‐O study authors (one of whom was BF), and reviews added to the present review were assessed by the authors (MK, ZC, and BF) with each paper being assessed by two reviewers. Any disagreements were resolved through discussion.

**TABLE 1:** AMSTAR‐2 gradings

AMSTAR‐2 items	Number of systematic reviews which failed to meet this criterion (in ascending order)
Appropriateness of meta‐analytical methods (item 11)	0/21 (0%)
Included studies described in adequate detail (item 8)	7/44 (15.9%)
Assessment of presence and likely impact of publication bias (item 15) [if MA was performed]	5/21 (23.8%)
Impact of risk of bias assessed in MA (item 12)	5/21 (23.8%)
Risk of bias assessed from individual studies being included in the review (item 9)	13/44 (29.5%)
Potential conflicts of interest of the review authors reported (item 16)	13/44 (29.5%)
Adequacy of the literature search (item 4)	16/44 (36.4%)
Research question and inclusion criteria included components of PICO (item 1)	16/44 (36.4%)
A satisfactory explanation and discussion of any heterogeneity observed (item 14)	17/44 (38.6%)
Consideration of risk of bias when interpreting the results of the review (item 13)	18/44 (40.9%)
Study selection performed in duplicate (item 5)	21/44 (47.7%)
Data extraction performed in duplicate (item 6)	29/44 (65.9%)
Justification for excluding individual studies (item 7)	31/44 (70.5%)
Selection of included study designs explained (item 3)	32/44 (72.7%)
Sources of funding reported for the individual included studies (item 10)	37/44 (84.1%)
Protocol registered before commencement of the review (item 2)	38/44 (86.4%)

### 2.5 Data synthesis

The qualitative data describing the study details extracted from the reviews are displayed in a PICO table (population, interventions, comparison type, outcome [types of outcome assessed in the MAs]) to demonstrate the extent of the current evidence and any possible gaps. All outcomes were included, and categorized into eight outcome groups (ED behaviors, ED psychopathology, remission/abstinence, weight/BMI, depression, other psychological outcomes, quality of life and percentage of dropouts). The definitions of “remission” and “abstinence” varied between the systematic reviews, and were often overlapping; thus, these two types of outcomes were grouped in one category.

The MA syntheses extracted from the included systematic reviews are presented in data tables, including information about the type of MA comparison, the sample studied and the results (statistical significance, effect sizes when the results are statistically significant [*p*‐value ≤ .05], and the number of included RCTs/synthesis). The emphasis of the reporting is on MAs that compared CBT against active controls. Summary tables of the syntheses including inactive controls are presented in the Supplementary material (S2).

## 3 RESULTS

### 3.1 Included reviews

**FIGURE 1:**
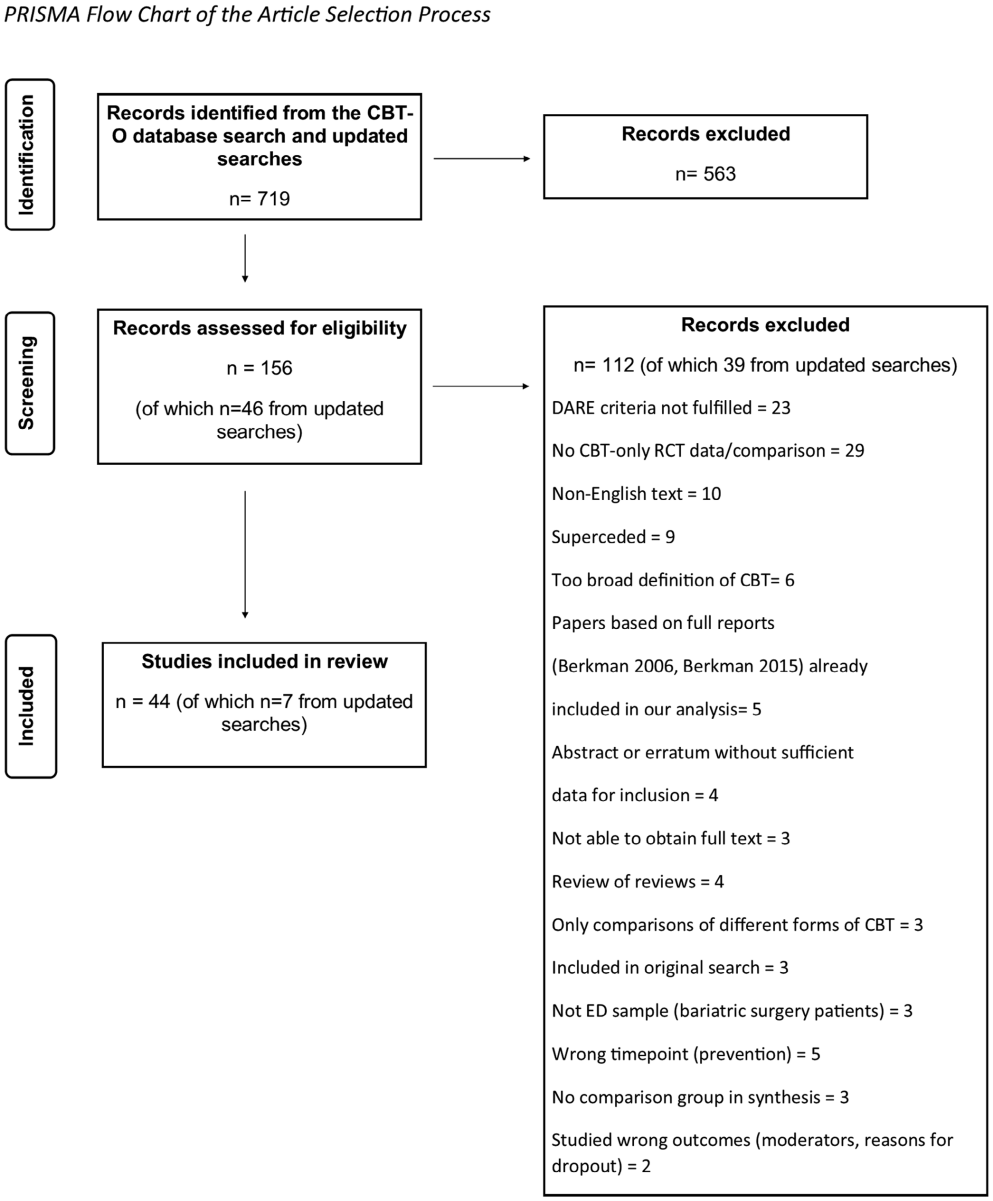
Flow chart of the article selection process

As can be seen in Figure 1, we included 44 systematic reviews, 37 from the original study and 7 further reviews from the updated searches. For a list of the included reviews, please see the References. The included reviews are presented in Table 2 together with a brief description of their characteristics using PICO criteria.

**TABLE 2:** Description of the systematic reviews presented using their PICO criteria

Review ID^a^	RCTs	Type of ED				Type of CBT						Comparison				Outcomes in the MA							
		BED	BN	AN	Other	High	Low	High and low	Generic	ED‐specific	Not specified	Psychotherapy	Pharmacotherapy	Other active	Inactive	ED psychopathology	ED behaviors	Abstinence/remission	Weight/BMI	Depression	Other psychological	Quality of life	Dropouts
Allen and Dalton (2011)	3	x	x				x			x				x	x								
**Atwood and Friedman** (2019)	**8**	**x**	**x**	**x**	**x**	**x**				**x**		**x**		**x**	**x**								
Beumont et al. (2004)	2	x		x		x					x	x		x									
**Berkman et al.** (2006)	**25**	**x**	**x**	**x**	**x**			**x**	**x**	**x**		**x**	**x**	**x**	**x**								
**Berkman et al.** (2015)	**11**	**x**						**x**	**x**	**x**		**x**			**x**		**x**	**x**					
Dahlenburg et al. (2019)	5	x	x	x	x	x				x		x		x									
Datta et al. (2022)	4	x	x	x		x			x		x	x			x								
De‐Bacco et al. (2017)	3	x	x	x		x				x	x	x			x								
de Jong et al. (2018)	4	x	x	x		x				x		x			x								
Flament et al. (2012)	1	x				x					x		x										
Ghaderi and Andersson (1999)	7		x			x					x	x	x	x	x								
**Ghaderi et al.** (2018)	**19**	**x**						**x**		**x**		**x**	**x**	**x**	**x**	**x**	**x**	**x**	**x**	**x**			
Giel et al. (2021)	2			x				x		x				x	x								
**Grenon et al.** (2017)	**22**	**x**	**x**	**x**	**x**			**x**			**x**	**x**	**x**	**x**	**x**	**x**	**x**	**x**					
Grenon et al. (2018)	28	x	x	x	x			x			x	x		x	x	x	x	x		x	x		
**Hay et al.** (2001)	**5**		**x**			**x**				**x**	**x**	**x**	**x**		**x**		**x**	**x**		**x**			**x**
**Hay et al.** (2009)	**39**	**x**	**x**		**x**			**x**	**x**	**x**		**x**	**x**	**x**	**x**		**x**	**x**	**x**	**x**	**x**		**x**
Hay and Claudino (2010)	10		x					x	x	x		x	x	x	x								
Hay et al. (2012)	1			x		x				x				x									
Hay (2013)	18	x	x	x	x			x		x	x	x		x	x								
Hay et al. (2014)	1			x		x				x		x											
**Hay et al.** (2015)	**4**			**x**		**x**			**x**	**x**		**x**		**x**	**x**	**x**		**x**	**x**	**x**	**x**		**x**
Hilbert et al. (2019)	29	x						x			x	x	x	x	x	x	x	x	x	x			x
Keel and Haedt (2008)	28	x	x	x	x			x			x	x	x	x	x								
**Linardon & Brennan** (2017)	**24**	**x**	**x**	**x**	**x**			**x**	**x**	**x**		**x**	**x**		**x**							**x**	
**Linardon, Fairburn, et al**. (2017)	**3**	**x**	**x**	**x**				**x**		**x**	**x**	**x**				**x**	**x**	**x**					
**Linardon et al.** (2017a)	**78**	**x**	**x**	**x**	**x**			**x**	**x**	**x**		**x**	**x**	**x**	**x**	**x**	**x**	**x**					
**Linardon et al.** (2017b)	**23**		**x**					**x**			**x**	**x**	**x**	**x**	**x**		**x**			**x**			
**Linardon**, **Hindle, & Brennan** (2017)	**98**	**x**	**x**	**x**	**x**			**x**	**x**	**x**		**x**	**x**	**x**	**x**								**x**
Linardon (2018)	28	x	x		x			x		x		x	x		x	x	x						
**Linardon et al.** (2019)	**23**	**x**	**x**		**x**			**x**			**x**	**x**		**x**	**x**						**x**		
Loucas et al. (2014)	6	x	x	x	x		x			x					x	x	x	x					
Miniati et al. (2018)	21	x	x	x	x			x		x	x	x		x	x								
Palavras et al. (2017)	10	x	x		x			x			x	x		x	x		x		x				x
Pittock and Mair (2010)	4			x		x					x	x		x	x								
Pittock et al. (2018)	3		x		x		x			x	x				x								
**Polnay et al**. (2014)	**8**		**x**			**x**					**x**	**x**		**x**	**x**		**x**	**x**		**x**			
Reas and Grilo (2008)	1	x				x					x		x										
**Solmi et al.** (2021)	**5**			**x**		**x**				**x**	**x**	**x**		**x**	**x**		**x**		**x**				**x**
**Svaldi et al.** (2019)	**28**		**x**					**x**			**x**	**x**	**x**	**x**	**x**		**x**	**x**		**x**			**x**
Thompson Brenner et al. (2003)	16		x			x					x	x		x	x								
**Van den Berg et al.** (2019)	**4**			**x**		**x**				**x**	**x**	**x**		**x**	**x**	**x**			**x**			**x**	
Vogel et al. (2021)	16	x	x	x	x			x			x	x		x	x								
Watson and Bulik (2013)	4			x		x					x	x		x									

Abbreviations: High, high intensity; low, low intensity; other, most often this was EDNOS; RCT, number of RCTs extracted from the review.

^a^ The reviews rated as moderate or high quality are bolded.

### 3.2 Quality of the reviews

Of the 44 reviews, 18 (40.9%) were graded as of high/moderate quality while 26 (59.1%) were graded as of low/critically low quality. Table 1 presents the AMSTAR‐2 items (Shea et al., 2017) and the number of reviews that failed to meet each criterion. The most common items that reviews failed to meet were: publishing a protocol prior to conducting the review, reporting the sources of funding and reporting the reason for the study designs selected for inclusion.

Inter‐rater reliability (calculated as % of agreement on individual AMSTAR items) was 74.5% between ZC and MK, and 81.3% between BF and MK.

### 3.3 Qualitative synthesis of the reviews

#### 3.3.1 Participants

Most reviews (*n* = 24, 54.5%) combined data collected from participants with different EDs (i.e., at least partly transdiagnostic samples, referred to as “mixed” from here on), and of these 18 (40.9%) included participants with eating disorder not otherwise specified (DSM IV EDNOS). Five (11.4%) included reviews were conducted exclusively with BED populations, seven (15.9%) exclusively with BN populations and eight (18.2%) exclusively with AN populations.

Twenty‐nine (65.9%) reviews included data from adult populations, whereas two (4.5%) reviews focused solely on young people (<18 years old) and none on older adults (≥65 years old). Eight (18.1%) reviews did not report the age of participants, and 19 (43.2%) reviews did not report the sex or gender of the participants. Eleven (44% of the reviews that reported sex) reviews included only female participants. Where men were included (*n* = 14, 56% of the reviews that reported sex), the percentage of men in the samples studied ranged between 2% and 41%. Other gender identities were not addressed in any of the reviews. Two reviews reported the ethnicity of some participants, but also included RCTs that did not report ethnicity. Participants in these two reviews were 57%–98% White ethnic groups. Sixteen reviews were high middle income countries (HMIC), while others did not report these data. Fifteen reviews mentioned comorbid conditions, but only four reviews reported and/or included any analysis of these conditions.

#### 3.3.2 Interventions

Twenty‐two (50%) reviews combined data from both high and low intensity CBT interventions (see Table 2). Nineteen (43.2%) included only high intensity (individual or group CBT). The three (6.8%) reviews studying exclusively low intensity CBT combined guided and unguided self‐help.

Often reviews did not explicitly state whether the CBT intervention was ED‐focused or generic CBT, and this had to be inferred. Ten (22.7%) reviews only included ED‐focused CBT interventions. The remainder included both ED‐focused and generic CBT or provided no information regarding CBT type.

#### 3.3.3 Comparison interventions

Most reviews (*n* = 34, 77.3%) combined data from RCTs that compared CBT to both active and inactive control groups (Table 2). Eight (18.1%) reviews included RCTs with only active comparisons and two reviews (4.5%) compared CBT exclusively with inactive comparators. The active comparator interventions included other forms of psychological treatments (e.g., interpersonal psychotherapy, behavioral treatment, supportive psychotherapy, or psychodynamic psychotherapy) and pharmacotherapy (most often antidepressant medication) amongst other active interventions.

#### 3.3.4 Types of outcome reported in the MAs

The most common outcome studied in the MAs was ED behaviors (16 MAs, 76.1% of all MAs), most commonly binge eating and/or purging. Abstinence and/or remission was reported in 13 (61.9%) MAs, operationalized as abstinence from key behavioral symptoms or remission from ED symptoms or ED diagnosis. The time frame for abstinence varied between the reviews and RCTs. ED psychopathology outcomes were studied in 10 (47.6%) MAs; most often as assessed by the Eating Disorder Examination Questionnaire EDE(−Q) (Fairburn & Beglin, 2008) or Eating Disorder Inventory (EDI) (Garner, 2004; Garner et al., 1983). Weight‐related outcomes (weight or BMI) were studied in seven (33.3%) MAs. Depressive symptoms were studied in nine (42.9%) MAs, dropout in eight (38.1%) MAs, other psychological outcomes (self‐esteem, self‐concept, general psychiatric functioning or interpersonal functioning) in four (19.0%) MAs and quality of life in two (9.5%) MAs.

Potential harms related to treatment were addressed in nine reviews but the lack of details in the RCTs prevented the synthesis and analysis of these.

Almost half of the reviews (*n* = 19, 43.2%) reported short and long term outcomes with only three (6.8%) reviews reporting exclusively on studies of long‐term outcome. Meta‐analytic syntheses at long term follow up were performed in five (23.8% of all MAs) reviews, all on high or mixed intensity CBT compared to active control conditions.

#### 3.3.5 Quantitative results from the meta‐analytic syntheses

Of the 44 reviews 21 (47.7%) included at least one meta‐analytic synthesis comparing CBT with a non‐CBT intervention or an inactive control. Tables 3, 4, 5, 6 present the findings from the MAs including active controls. Effect sizes are reported for the syntheses with statistically significant effects that included more than 1 RCT in comparison with an active control. If more than one synthesis studied a similar type of comparison, the effect size is reported for the synthesis that included more RCTs, or if syntheses included a similar number of RCTs, effect sizes from both are reported. The effect sizes are presented as they were reported in the original paper.

**TABLE 3:** Summary table of MA syntheses comparing CBT to a variety of active controls pooled together

CBT intensity		Control	ED type	Outcomes			
				ED behaviors	ED psychopathology	Abstinence/remission	Weight
High		Various active controls	BED	**Y (9 RCTs, g= .18),** N(2 RCTs)	**Y (8 RCTs, g=.17),** N(2 RCTS)	N (1‐5 RCTs)	
			BN	**Y (20‐25 RCTs, g=.21)**	**Y (16‐18 RCTs, g=.20)**	**Y (15 RCTs, OR=1.49)**, N (14 RCTs)^1^	
			AN		N (2‐10 RCTs)	N (2 RCTs)	N (2 RCTs)
			Mixed		**Y (15 RCTs, g=.18‐.33)** ^**X**^		
	CBT‐E		BN	**Y (3 RCTs, g=.52)**	**Y (4 RCTs, g=.52)**	N (3 RCTs)	
			AN		N (3 RCTs)		N (3 RCTs)
			Mixed		N (5 RCTs)^X^		
	CBT‐BN		BN	N (4 RCTs)	**Y (3 RCTs, g=.53)**	N (4 RCTs)	
			Mixed		**Y (17 RCTs, g=.23‐.27)** ^**X**^		
	CBT‐BN/E		BED	N (1 RCT)	N (1 RCT)	N (1 RCT)	
			BN	**Y (7 RCTs, g=.42)**	**Y (7 RCTs, g=.53)**	**Y (7 RCTs, OR=2.08)**	
	“adapted” CBT‐BN		BED	N (2 RCTs)	N (2 RCTs)	N (2 RCTs)	
			BN	N (11 RCTs)	N (10 RCTs)	N (7 RCTs)	
	group CBT		BED	N (6 RCTs)	N (7 RCTs)	N (4 RCTs)	
			BN	N (5 RCTs)	N (2 RCTs)	N (1 RCT)	
High and low			BED	N (5 RCTs)	**Y (6 RCTs, g=.27)**, N (4 RCTs)^X 2^	N (2 RCTs)	
			BN	N (7 RCTs)	**Y (12 RCTs, g= .21‐.27)** ^**X**^, **N(1‐12 RCTs)** ^**X 3**^	N (1 RCT)	
			Mixed	**Y (18 RCTs, g=.29)** ^**X**^	**Y (22 RCTs, g=.24‐.31)** ^**X**^		
Low			BED	N (4 RCTs)	N (5 RCTs)	N (4 RCTs)	
			Mixed		**Y (7 RCTs, g= .34, .39)** ^**X**^, N (7 RCTs)^X 4^		

CBT intensity	Control	ED type	Outcomes			
			Depression	Other psychological	Quality of life	Dropouts
High	Various active controls	BED				N (10 RCTs)
		BN				N (23 RCTs)
		AN	N (1 RCT)	N(1 RCT) ^5^	N (3 RCTs, CBT‐E)	N (9 RCTs)
		Mixed				N (44 RCTs)
High and low		BED		N (6 RCTs) ^6^		
		BN		N (8 RCTs) ^7^		
		Mixed			**Y (9 RCTs, g=.36)**, N (3 RCTs) ^8^	

*Note*: Where the results are difficult to interpret, the differences between the syntheses are explained, and the outcomes categorized under “Other psychological” are specified (see numbers 1–8). The number of RCTs reported in parentheses refers to the number of RCTs in the synthesis/‐es in question. The statistically significant results (Y) are bolded. Y = a statistically significant result in favor of CBT, N = no statistically significant difference. SMD = standardized mean difference. g = Hedge's g. OR = risk ratio. For a detailed definition of “adapted CBT‐BN”, please see Linardon et al. 2017a. (1) Many overlapping RCTs in the comparisons; Y: individual and group, N: only individual, close to statistical significance (*p* = .062), see Linardon et al. 2017a. (2) N: dietary restraint, shape concern, weight concern, Y: cognitive symptoms. (3) Y: weight concern, dietary restraint, N: shape concern (12 RCTs), cognitive symptoms (1 RCT). (4) Y: shape concern, weight concern; N: dietary restraint. (5) Outcome: general psychiatric score. (6) Outcome: self‐esteem. (7) Outcome: self‐esteem. (8) Y: subjective QoL, N: health‐related QoL. ^X^The result is from a low/critically low quality review.

**TABLE 4:** Summary table of MA syntheses comparing CBT to particular active control interventions individually (ED‐specific outcomes)

CBT intensity	Control	ED type	Outcomes				
			ED behaviors	ED psychopathology	Abstinence/remission	Weight	
High	Behavioral weight loss treatment	BED	**Y (4 RCTs, MD= 2.04** ^**X**^ **, SMD=.31)**		N (2 RCTs)	N (4 RCTs)
		Mixed	**Y (5 RCTs, g=.30)**	N (5 RCTs)	N (4 RCTs)		
	Supportive therapy	Mixed	N (6 RCTs)	N (4 RCTs)	N (2 RCTs)		
	Pharmacological	BED	N (2 RCTs)	**Y (2 RCTs, g=.73)**			
		BN	N (1 ‐ 4 RCTs)	N (4 RCTs)	N (3‐5 RCTs)		
	Various psychotherapies	BED	**Y (2 RCTs, SMD=.21)** ^**X**^ **,** N (1 RCTs) ^**1**^	N (3 RCTs) ^X^	N (1‐3 ^X^ RCTs)	N (1‐3 RCTs)	
		BN	**Y (15 RCTs, g=.33**), N (8‐15 RCTs) ^**2**^		**Y (7 RCTs, RR= .83)**	N (5 RCTs)	
		AN		N (2 RCTs)	N (2 RCTs)	N (2 RCTs)	
		EDNOS	N (1 RCT)				
		Mixed	**Y (15 RCTs, SMD=.21),** N (5^X^ RCTs) ^**3**^	**Y (7‐11 RCTs, g=.31)** ^**X**^	N (7 ^X^‐10 RCTs)	**control (11 RCTs, SMD=.18)**	
	Interpersonal psychotherapy	BED	N (1 RCT)^X^	N (1 RCT)^X^	N (1 RCT)^X^	N (1 RCT)^X^	
		Mixed	**Y (6 RCTs, g=.24)**	**Y (6** ^**X**^ **‐7 RCTs, g=.32)**	N (6 RCTs)		
	Behavioral psychotherapy	Mixed	N (8 RCTs)	N (7 RCTs)	N (5 RCTs)		
	Psychodynamic psychotherapy	BED	N (1 RCT)^X^	N (1 RCT)^X^	N (1 RCT)^X^	N (1 RCT)^X^	
	Humanistic psychotherapy	BED		N (1 RCT)^X^	**Y (1 RCT)** ^**X**^	N (1 RCT)^X^	
	Group psychotherapy	BED		N (3 RCTs)	N (3 RCTs)		
		BN	N (1 RCT)	N (1 RCT)			
		Mixed		N (4 RCTs)			
	Thirdwave psychotherapies	BED	N (2 RCTs)	**Y (1 RCT),** N (2 RCTs) ^**4**^	N (1 RCT)		
		Mixed	N (3 RCTs)	N (2‐3 RCTs)	N (3 RCTs)		
High and low	Behavioral weight loss treatment	BED	**Y(4 RCTS, SMD=.27)**		N (4 RCTs)	N (4 RCTs)	
	Various psychotherapies	BN	**Y (13 RCTs, g=.30),** N (17 RCTs) ^**5**^				
	Interpersonal psychotherapy	BED	N (2 RCTs)		N (2 RCTs)	N (2 RCTs)	
Low	Bibliotherapy	BN	N (1 RCT)^X^	N (1‐2 RCTs)^X^	N (1‐2 RCTs)^X^		
	Self‐compassion training	BED	N (1 RCT)^X^	N (1 RCT)^X^		N (1 RCT)^X^	
	Various psychotherapies	BN	N (2‐3 RCTs)				

*Note (for Tables* *4* *and* *5* *)*: Where the results are difficult to interpret, the differences between the syntheses are explained, and the outcomes categorized under “Other psychological” are specified (see numbers 1–7 in Table 4, and numbers 1–6 in Table 5). The syntheses that excluded poor quality RCTs are underlined. The number of RCTs reported in parentheses refers to the number of RCTs in the synthesis/‐es in question. The statistically significant results (Y) are bolded. Y = a statistically significant result in favor of CBT, N = no statistically significant differences, control = a statistically significant result in favor of control group. g = Hedge's *g*. MD = mean difference. RR = risk ratio. SMD = standardized mean difference. (1) Y = binge‐eating days, N = mean bulimic symptoms (2) Y = binge frequency, N = purge frequency (15 RCTs), mean bulimic symptoms (8 RCTs), (3) Y = mean bulimic symptoms, N = binge/purge frequency, (4) Y & N: both studied similar ED psychopathology outcomes, (5) Y = binge frequency, N = purge frequency. ^X^ = the result is from a low/critically low quality review.

**TABLE 5:** Summary table of MA syntheses comparing CBT to particular active control interventions individually (non‐ED‐specific outcomes)

CBT intensity	Control	ED type	Outcomes		
			Depression	Other psychological	Dropouts
High	Behavioral weight loss treatment	BED	N (4 RCTs)	N (1 RCT)^1^	N (3 RCTs)
	Pharmacological	BN	N (3 RCTs)		**Y (4 RCTs**, **RR = ‐2.18)**
	Various psychotherapies	BED	N (1‐2^X^ RCTs)	N (1 RCT)^2^	N (1–4 ^X^ RCTs)
		BN	**Y (15 RCTs**, **g = .25)**, N (7 RCTs))^3^	N (4 ^4^ ‐5^5^RCTs)	N (1–8 RCTs)
		AN	N (1 RCT)	N (1 RCT) ^6^	N (2 RCTs)
		Mixed	N (1–13 RCTs)	N (4^X^‐7 RCTs) ^7^	N (14 RCTs)
	Interpersonal psychotherapy	BED			N (1 RCT)^X^
	MANTRA	AN			N (NR RCTs)
	Psychodynamic	BED	N (1 RCT)^X^		N (1 RCT)^X^
		AN			**Y (NR RCTs**, **OR = .54)**
	Familybased psychoterapy	AN			N (NR RCTs)
	Humanistic therapy	BED	**Y (1 RCT)** ^**X**^		N (1 RCT)^X^
High and low	Behavioral weight loss treatment	BED	N (3 RCTs)		
	Various psychotherapies	BN	N (18 RCTs)		
Low	Self‐compassion training	BED	N (1 RCT)^X^		N (1 RCT)^X^
	Mixed psychotherapy	BN	N (1‐2RCTs)		

*Note*: (1) outcome = interpersonal functioning, (2) outcome = interpersonal functioning and general psychiatric score, (3) N (7 RCTs) close to statistical significance (see Hay et al., 2009; Linardon et al. 2017b), (4) outcome = interpersonal functioning, (5) outcome = general psychiatric score, (6) outcome = general psychiatric score, (7) outcome = interpersonal functioning, self‐concept, general psychiatric score. ^X^ = the result is from a low/critically low quality review.

Abbreviation: NR, not reported.

**TABLE 6:** A summary table of MA syntheses conducted at long follow‐up (1 year or over)

CBT intensity	Control	ED type	ED behaviors	ED psychopathology	Abstinence/remission	Weight/BMI	Depression	Other psychological	*Dropouts*
High	Various active	BED	N (4 RCTs)	N (4 RCTs)	N (4 RCTs)				
		BN	**Y (10 RCTs**, **g = .31)**	N (9 RCTs)	N (6 RCTs)				
		AN		N (2–6 RCTs)	N (1 RCT)	N (2 RCTs)	N (1 RCT)	N (1 RCT) ^**1**^	
	Behavioral weight loss treatment	BED	N (3 RCTs)^X^			N (3 RCTs)^X^			N (4 RCTs)^X^
	Pharmacological	BED	N (2 RCTs)	**Y (3 RCTs**, **g = .99)**	**Y (1 RCT)**				
		BN	N (1 RCT)	N (1 RCT)	N (1 RCT)				
	Various psychotherapies	AN		N (2 RCTs)	N (1 RCT)	N (2 RCTs)	N (1 RCT)	N (1 RCT) ^**2**^	
	Family‐based psychotherapy	AN	N (NR RCTs)			N (NR RCTs)			
	MANTRA	AN	N (NR RCTs)			N (NR RCTs)			
	Psychodynamic psychotherapy	AN	N (NR RCTs)			N (NR RCTs)			
	Treatment as usual	AN	N (NR RCTs)			N (NR RCTs)			
High and low	Interpersonal psychotherapy	BED	N (2 RCTs)		N (2 RCTs)	N (2 RCTs)			
	Behavioral weight loss treatment	BED	**Y (3 RCTs**, **SMD = .24)**		**Y (3 RCTs**, **RD = .13)**	N (3 RCTs)	N (2 RCTs)		

*Note*: The outcomes categorized under “Other psychological” are specified ^(see numbers 1–2)^. The syntheses that excluded poor quality RCTs are underlined. The number of RCTs reported in parentheses refers to the number of RCTs in the synthesis/−es in question. The statistically significant results (Y) are bolded. Y = a statistically significant result in favor of CBT, N = no statistically significant differences. g = Hedge's *g*. SMD = standardized mean difference. RD = risk difference. (1) outcome: general psychiatric score, (2) outcome: general psychiatric score. ^X^ = the result is from a low/ critically low quality review. NR = not reported.

Table 3 presents the data from reviews comparing CBT to any active control group. Tables 4 and 5 present the data comparing CBT to specific active controls groups and Table 6 presents the MAs of long‐term follow‐up data. Data from reviews which compared CBT to inactive control groups are presented in Tables 7a and 7b in the Supplementary material. Of the reviews with a MA, 16 (76.1%) were rated as moderate or high quality. Results extracted from the critically low/low quality reviews are marked in the tables.

The results from MA syntheses that compared CBT to active and inactive controls pooled together are reported in the summary of results but not in the tables.

#### 3.3.6 Quality of RCTs in the MAs

Only three of the MAs (14.2%) were conducted solely with RCTs rated as having low or moderate risk of bias. Ten (47.6%) reviews assessed the moderating effect of higher and lower quality RCTs and reported that the quality of the RCTs did not moderate effects reported. Six (28.6%) reviews assessed RCT quality but neither analyzed nor discussed its effects on their reported results. Four (19.0%) reviews did not assess RCT quality.

#### 3.3.7 Summary of results

##### CBT compared to active controls

High intensity CBT was more effective compared to mixed active controls in reducing ED behaviors and psychopathology (see Table 3). It was also more effective than behavioral weight loss in reducing ED behaviors, and more effective than interpersonal psychotherapy in reducing ED behaviors and psychopathology. The results are mixed when CBT was compared against pharmacological interventions and a mixed group of various psychotherapies. CBT has not been shown to be more effective than behavioral or supportive therapy on any of the outcomes. Comparisons against other forms of psychotherapy have included few RCTs. (see Tables 4, 5). Aside from ED specific outcomes, the only effects favoring CBT against active controls were for depression and number of dropouts and only against certain specific control interventions (see Table 5).

In syntheses that pooled active and inactive controls (not in the data tables), high intensity CBT was effective in increasing self‐esteem (9–14 RCTs per synthesis). There was also some support for high and low intensity CBT leading to improvements in quality of life outcomes in both those with BN and in mixed ED samples (3–13 RCTs/synthesis), but no significant effect for those with BED (3–4 RCTs/synthesis).

##### Effects of different forms of CBT

**Low and high intensity CBT.** We identified a large evidence base for high intensity CBT. Low intensity CBT (pure self‐help or guided self‐help) MAs with active controls did not include the following: the study of AN populations, reports on long term outcomes and an examination of the effect of the quality of the RCTs. One MA synthesis found an effect in favor of low intensity CBT on certain ED psychopathology features (see Table 3, 4). In MA syntheses where active and inactive controls were pooled, the only significant favorable effects of low intensity CBT were on quality of life (3–5 RCTs/synthesis).

**Group‐based CBT.** We found evidence that group CBT is more effective than inactive control conditions but not more effective than active control conditions for BN and BED populations (see Table 3). There are no data for AN. No effect favoring group CBT was found when compared to active and inactive interventions pooled together (quality of life, three RCTs/synthesis).

**Specific CBT protocols.** The data support CBT‐BN (Fairburn et al., 1993) and its enhanced transdiagnostic form (CBT‐E) (Fairburn et al., 2008) as more effective than active and inactive control conditions for mixed diagnostic and BN populations with regard to ED behavior and psychopathology, although not all syntheses produced consistent results. CBT‐BN was not favored when it was described as “adapted” (see Linardon et al. 2017a, see Table 3). CBT‐BN/E has shown a favorable effect on reported quality of life (six RCTs/synthesis) but no effect on health‐related quality of life (two RCTs/synthesis) in syntheses that pooled active and inactive control interventions.

##### Effects across various eating disorder presentations

As we have seen, benefits for CBT have been reported on a variety of outcomes in those with BED, BN, and for mixed diagnostic groups, but no significant effects have been demonstrated for CBT as compared to other active treatments in those with AN, other than one MA synthesis that found a statistically significant effect in favor of CBT in the percentage of dropouts from treatment.

##### Effects across various demographic groups

The systematic review data is almost exclusively generated from adults, predominantly women, who are white and live in HMI countries. Comorbid conditions, with the exception of depression, have received relatively little attention in the MA syntheses. There are a lack of data to explore whether sex and gender, ethnicity, country of origin or age moderates the effectiveness of CBT on ED outcomes.

##### Longer term effects

The effectiveness of CBT at follow up of 12 month or longer is unclear (see Table 6) with some reviews finding positive effects on ED behaviors and psychopathology in BED and BN populations, while others did not.

##### Range of outcomes

In comparison to active control conditions, the strongest evidence for CBT has been found for the outcomes of ED behaviors and ED psychopathology.

## 4 DISCUSSION

The current overview of the evidence supporting CBT for EDs aimed to provide a critical synthesis of the large and growing systematic review literature in the field. It was undertaken with the explicit aim of synthesizing evidence drawn from previously conducted systematic reviews to identify the extent and strength of the evidence for CBT, identify any gaps in the evidence and to examine the quality of the systematic reviews from which it is drawn. By undertaking a review of the evidence at this higher level of generalization, we aimed to transcend the more limited scope of individual systematic reviews.

Consistent with current guidelines (Hilbert et al., 2017), our review confirmed that CBT produces benefits for people with symptoms of binge eating and/or purging (generally those with BED, BN and EDNOS/OSFED). More particularly, it made clear that CBT is most effective in producing good outcomes for these groups on both ED behavior and psychopathology and, to a lesser extent, abstinence/remission, when delivered face‐to‐face on an individual basis. The review with its explicit aims to assess systematic review evidence guided by a number of specific research aims also highlighted significant gaps in the evidence that need to be addressed in future research.

First, the current evidence base shows statistically significant effects for individually delivered high intensity CBT over a mixed group of active control interventions (as well as over certain specific psychological and psychotherapeutic approaches) with regard to certain ED‐specific outcomes, although the effect sizes are small. However, overall, the current evidence base does not fully support CBT as generally more effective than other specific psychotherapeutic treatments. Further investigation of, for whom and under what circumstances, this form of CBT might be the treatment of choice has the potential to greatly enhance clinical benefit for patients.

Second, as regards the form of CBT, our overview of systematic reviews shows that low intensity CBT has received significantly less attention than high intensity CBT. The evidence supporting low intensity CBT, as well as group CBT, was relatively weak as there was only support for these forms of CBT when compared to inactive control interventions. A possible exception was for a particular form of low intensity CBT, guided self‐help, which has the potential to be made much more widely available with the possibility of helping to bridge the well‐documented treatment gap in EDs (Kazdin et al., 2017). CBT in guided self‐help form produced benefits for certain features of ED psychopathology even when compared to active interventions, and benefits to quality of life in comparison to active and inactive controls pooled together.

The MA syntheses studying specific manualized approaches supported CBT‐BN (Fairburn et al., 1993) and CBT‐E (Fairburn et al., 2008), but not the “adapted” approaches. However, the data is limited and not all syntheses produce consistent results. Further data on whether some approaches are more potent than others for all patients, or for particular subgroups of patients would provide potentially valuable evidence to guide clinicians about how to help patients gain the most from CBT.

Third, there was very limited evidence supporting CBT for those who are significantly low weight and generally receive a diagnosis of AN, and no evidence to support it as more effective than other active ED focused psychological treatments. Thus, while CBT in its present form can be used transdiagnostically, its effects for those who are low weight are less positive than for some groups with EDs. This finding points to a pressing need for further treatment innovation to improve outcomes for this group.

Fourth, the insufficient reported data concerning the age, sex and gender, culture or country of residence of those being treated, calls into question the generalizability of the effects of CBT across various populations. Also, considering the high levels of comorbid conditions in those with EDs, the lack of data on the effects of CBT on those with EDs and comorbid conditions limits generalizability to the full range of clinical presentations. These gaps in the data limit the evidence‐based guidance that can be provided to clinicians and point to a need to examine the effectiveness of CBT in a wider group of patients. One recent systematic review did consider a variety of demographic and comorbid conditions as moderators of the effect of CBT (Linardon, de la Piedad Garcia, & Brennan, 2017) but the findings were inconclusive, and the review author identified the need for more research on the mediators and moderators of the effectiveness of CBT for EDs. Further understanding of the moderators of treatment effects would allow better matching of treatments and perhaps highlight the need for further treatment development for some sub‐groups.

Fifth, the longer‐term effects of CBT beyond 12 month follow up are not clear. This is because of the relatively few studies of longer term follow up and the inconsistency of the existing results.

Sixth, our review highlighted the limited range of outcomes reported for CBT, and the lack of consensus on how they are operationalized in the syntheses. Other than ED specific outcomes, there is relatively little evidence of any other effects produced by CBT for EDs. For example, there is limited data on impairment of functioning—an important omission when considering that impairment is often a key factor in making a diagnosis of a disorder and in patients seeking treatment.

An important strength of our study was that we assessed the quality of the reviews being synthesized using AMSTAR, a widely used instrument for critically appraising systematic reviews. We found that the quality of the reviews included varied, as did the quality of the RCTS included in these reviews. The most common shortcomings of the systematic reviews were not registering a protocol before commencing the review, not reporting the funding sources of the RCTs included and, importantly, not providing an explanation for the selection of studies reviewed. Most of the reviews did not exclude poor quality RCTs from their MA syntheses, although many did study the moderating effect of their quality.

A limitation of our report is that we have only provided the main quantitative results and have left out some of the more detailed information that is usually included when reporting a MA. Data were only extracted at the review level and so some data from relevant RCTs were not explicitly represented. We excluded reviews not published in English (*n* = 10), which might have addressed one of the evidence gaps identified, namely few RCTs performed in settings outside of Western cultures. We also excluded consideration of the newly recognized feeding disorders in DSM 5, and studies that directly compare different forms of CBT, limiting our ability to draw conclusions about the treatment of these feeding disorders and the efficacy of different forms of CBT. The MAs studied various outcome variables and diagnostic groups, and the operationalization of definitions of variables and subthreshold disorders was not always explicit and varied between the systematic reviews. This was particularly an issue with the outcome of abstinence/remission as noted earlier. To synthesize a large amount of sometimes disparate data, we had to combine outcomes in a way that may obscure some finer grained details. Lastly, a single researcher performed the data extraction and synthesis.

In assessing the qualitative and meta‐analytic syntheses in our overview it is important to remember that many include the same RCTs; i.e. certain RCTs are “recycled” and studied in different syntheses. We suggest further study of the quality and generalizability of any possible RCTs that might play a key role in determining the statistical significance of the various MA syntheses.

In summary, while the results of our current overview of the systematic review evidence provide support for some particular forms of CBT for certain ED presentations, an important finding was that there are major gaps in the current evidence that need to be addressed in the future. One of the most important and pressing concerns is the limited data on those who receive a diagnosis of AN. Although currently there are three main approaches recommended by the clinical guidelines (e.g., NICE, 2017), there is limited evidence to support CBT as the treatment of choice and there is an urgent need to address the relatively poor outcomes to date of treatment for this group (Mulkens & Waller, 2021). In addressing the gaps in the existing evidence, there is a need to ensure that future research is conducted in careful alignment with quality criteria to produce RCTs with low risk of bias and systematic reviews of high quality. We have identified that the quality of the synthesized evidence base to date has not been uniformly high.

It is important to note that while our overview highlights limited evidence or the absence of certain evidence, it does not constitute evidence against CBT, but rather points to areas for future innovation and research. Target areas for future high quality research should include: a better understanding of the potential generalizability of CBT by studying the moderating effects of age, sex and gender, ethnicity, culture country of origin and the presence of comorbid conditions upon the effectiveness of CBT for EDs and assessing a wider range of outcomes for CBT by including a systematic study of clinical impairment and quality of life. Further targets for innovation are the development and testing of new approaches to benefit those groups, particularly those who are significantly low in weight, where outcome to date has been relatively poor and the further development, implementation and testing of potentially more widely available low intensity interventions that might bridge the well documented treatment gap in EDs.

## AUTHOR CONTRIBUTIONS

**Milla Kaidesoja:** Conceptualization; data curation; investigation; methodology; project administration; visualization; writing – original draft; writing – review and editing. **Zafra Cooper:** Conceptualization; investigation; methodology; supervision; writing – original draft; writing – review and editing. **Beth Fordham:** Conceptualization; investigation; methodology; resources; supervision; writing – original draft; writing – review and editing.

## CONFLICT OF INTEREST

No conflicts of interest. No specific funding was received for this work.

## Supporting information


**Appendix S1.** Supporting information.

**Appendix S2.** Supporting information.

**Appendix S3.** Supporting information.

**Appendix S4.** Supporting information.

## Data Availability

The data that support the findings of this study are available from the corresponding author upon reasonable request.

## References

[eat23831-bib-0001] Allen, S. , & Dalton, W. T. (2011). Treatment of eating disorders in primary care: A systematic review. Journal of Health Psychology, 16(8), 1165–1176. 10.1177/1359105311402244 21459921

[eat23831-bib-0002] Atwood, M. , & Friedman, A. (2019). A systematic review of enhanced cognitive behavioral therapy (CBT‐E) for eating disorders. International Journal of Eating Disorders, 53, 311–330. 10.1002/eat.23206 31840285

[eat23831-bib-0003] Beumont, P. , Beumont, R. , Hay, P. , Beumont, D. , Birmingham, L. , Derham, H. , Jordan, A. , Kohn, M. , McDermott, B. , Marks, P. , Mitchell, J. , Paxton, S. , Surgenor, L. , Thornton, C. , Wakefield, A. , & Weigall, S. (2004). Australian and New Zealand clinical practice guidelines for the treatment of anorexia nervosa. Focus, 3(4),1440–1614. 10.1080/j.1440-1614.2004.01449.x 15324328

[eat23831-bib-0004] Berkman, N. D. , Brownley, K. A. , Peat, C. M. , Lohr, K. N. , Cullen, K. E. , Morgan, L. C. , Bann, C. M. , Wallace, I. F. , & Bulik, C. M. (2015). Management and outcomes of binge‐eating disorder. Comparative effectiveness review No. 160. (prepared by the RTI International–University of North Carolina Evidence‐based Practice Center under contract No. 290–2012‐00008‐I.) AHRQ publication No. 15(16)‐EHC030‐EF. Agency for Healthcare Research and Quality www.effectivehealthcare.ahrq.gov/reports/final.cfm 26764442

[eat23831-bib-0005] Berkman, N.D. , Bulik, C.M. , Brownley, K.A. , Lohr, K.N. , Sedway, J.A. , Rooks, A. , & Gartlehner, G. (2006). Management of Eating Disorders. Evidence report/technology assessment No. 135. (Prepared by the RTI International‐University of North Carolina Evidence‐Based Practice Center under contract No. 290‐02‐0016.) AHRQ Publication No. 06‐E010. Rockville, MD: Agency for Healthcare Research and Quality.

[eat23831-bib-0006] Dahlenburg, S. C. , Gleaves, D. H. , & Hutchinson, A. D. (2019). Treatment outcome research of enhanced cognitive behaviour therapy for eating disorders: A systematic review with narrative and meta‐analytic synthesis. Eating Disorders, 27(5), 482–502. 10.1080/10640266.2018.1560240 30632926

[eat23831-bib-0007] Datta, N. , Matheson, B. E. , Citron, K. , Van Wye, E. M. , & Lock, J. D. (2022). Evidence based update on psychosocial treatments for eating disorders in children and adolescents. Journal of Clinical Child & Adolescent Psychology, 1‐12, 1–12. 10.1080/15374416.2022.2109650 35950931

[eat23831-bib-0008] De‐Bacco, C. , Marzola, E. , Fassino, S. , & Abbate‐Daga, G. (2017). Psychodynamic psychotherapies for feeding and eating disorders. Minerva Psichiatrica, 58(3), 162–180. 10.23736/S0391-1772.17.01936-7

[eat23831-bib-0009] de Jong, M. , Schoorl, M. , & Hoek, H. W. (2018). Enhanced cognitive behavioral therapy for patients with eating disorders: A systematic review. Current Opinion in Psychiatry, 31, 436–444. 10.1097/YCO.0000000000000452 30188385 PMC6181276

[eat23831-bib-0010] Flament, M. F. , Bissada, H. , & Spettigue, W. (2012). Evidence‐based pharmacotherapy of eating disorders. International Journal of Neuropsychopharmacology, 15, 189–207. 10.1017/S1461145711000381 21414249

[eat23831-bib-0011] Ghaderi, A. , & Andersson, G. (1999). Meta‐analysis of CBT for Bulimia Nervosa: Investigating the effects using DSM‐III‐R and DSM‐IV criteria. Scandinavian Journal of Behavioural Therapy, 28(2), 79–87.

[eat23831-bib-0012] Ghaderi, A. , Odeberg, J. , Gustafsson, S. , Råstam, M. , Brolund, A. , Pettersson, A. , & Parling, T. (2018). Psychological, pharmacological, and combined treatments for binge eating disorder: A systematic review and meta‐analysis. PeerJ, 6, e5113. 10.7717/peerj.5113 29942715 PMC6015752

[eat23831-bib-0013] Giel, K. E. , Behrens, S. C. , Schag, K. , Martus, P. , Herpertz, S. , Hofmann, T. , Skoda, E. M. , Voderholzer, U. , Wietersheim, J. , Wild, B. , Zeeck, A. , Schmidt, U. , Zipfel, S. , & Junne, F. (2021). Efficacy of post‐inpatient aftercare treatments for anorexia nervosa: A systematic review of randomized controlled trials. Journal of Eating Disorders, 9(1), 1–13. 10.1186/s40337-021-00487-5 34654471 PMC8518230

[eat23831-bib-0014] Grenon, R. , Carlucci, S. , Brugnera, A. , Schwartze, D. , Hammond, N. , Ivanova, I. , Mcquaid, N. , Proulx, G. , & Tasca, G. A. (2018). Psychotherapy for eating disorders: A meta‐analysis of direct comparisons. Psychotherapy Research, 29(7), 833–845. 10.1080/10503307.2018.1489162 29958509

[eat23831-bib-0015] Grenon, R. , Schwartze, D. , Hammond, N. , Ivanova, I. , Mcquaid, N. , Proulx, G. , & Tasca, G. A. (2017). Group psychotherapy for eating disorders: A meta‐analysis. International Journal of Eating Disorders, 50, 997–1013. 10.1002/eat.22744 28771758

[eat23831-bib-0017] Hay, P. (2013). A systematic review of evidence for psychological treatments in eating disorders: 2005‐2012. International Journal of Eating Disorders, 46, 462–469. 10.1002/eat.22103 23658093

[eat23831-bib-0018] Hay, P. P. J. , Bacaltchuk, J. , Stefano, S. , & Kashyap, P. (2009). Psychological treatments for bulimia nervosa and binging. Cochrane Database of Systematic Reviews, 4. 10.1002/14651858.CD000562.pub3 PMC7034415 19821271

[eat23831-bib-0072] Hay, P. J., & Claudino, A. M. (2010). Bulimia nervosa. BMJ Clinical Evidence, 2010, 1009. PMC3275326 21418667

[eat23831-bib-0020] Hay, P. P. J. , Claudino, A. M. , & Kaio, M. H. (2001). Antidepressants versus psychological treatments and their combination for bulimia nervosa. Cochrane Database of Systematic Reviews, 4. 10.1002/14651858.CD003385 PMC6999807 11687197

[eat23831-bib-0021] Hay, P. J. , Claudino, A. M. , Touyz, S. , & Abd Elbaky, G. (2015). Individual psychological therapy in the outpatient treatment of adults with anorexia nervosa. Cochrane Database of Systematic Reviews, 7. 10.1002/14651858.CD003909.pub2 PMC6491116 26212713

[eat23831-bib-0022] Hay, P. , Chinn, D. , Forbes, D. , Madden, S. , Newton, R. , Sugenor, L. , Touyz, S. , & Ward, W. (2014). Royal Australian and New Zealand College of Psychiatrists clinical practice guidelines for the treatment of eating disorders. Australian & New Zealand Journal of Psychiatry, 48(11), 977–1008. 25351912 10.1177/0004867414555814

[eat23831-bib-0071] Hay, P. J., Touyz, S., & Sud, R. (2012). Treatment for severe and enduring anorexia nervosa: a review. Australian & New Zealand Journal of Psychiatry, 46(12), 1136–1144. https://doi.org.10.1177/0004867412450469 10.1177/0004867412450469 22696548

[eat23831-bib-0023] Hilbert, A. , Petroff, D. , Herpertz, S. , Pietrowsky, R. , Tuschen‐Caffier, B. , Vock, S. , & Schmidt, R. (2019). Meta‐analysis of the effectiveness of psychological and medical treatments for binge‐eating disorder. Journal of Consulting and Clinical Psychology, 87(1), 91–105. 10.1037/ccp0000358 30570304

[eat23831-bib-0024] Keel, P. K. , & Haedt, A. (2008). Evidence‐based psychosocial treatments for eating problems and eating disorders. Journal of Clinical Child & Adolescent Psychology, 37(1), 39–61. 10.1080/15374410701817832 18444053

[eat23831-bib-0025] Linardon, J. (2018). Meta‐analysis of the effects of cognitive behavioral therapy on the core eating disorder maintaining mechanisms: Implications for mechanisms of therapeutic change. Cognitive Behaviour Therapy, 47(2), 107–125. 10.1080/16506073.2018.1427785 29378481

[eat23831-bib-0026] Linardon, J. , & Brennan, L. (2017). The effects of CBT for eating disorders on quality of life: A meta‐analysis. International Journal of Eating Disorders, 50, 715–730. 10.1002/eat.22719 28430364

[eat23831-bib-0027] Linardon, J. , Fairburn, C. G. , Fitzsimmons‐Craft, E. E. , Wilfley, D. E. , & Brennan, L. (2017). The empirical status of the third‐wave behaviour therapies for the treatment of eating disorders: A systematic review. Clinical Psychology Review, 58, 125–140. 29089145 10.1016/j.cpr.2017.10.005

[eat23831-bib-0028] Linardon, J. , Wade, T. D. , de la Piedad, X. , & Brennan, L. (2017a). The effectiveness of CBT for eating disorders: A systematic review and meta‐analysis. Journal of Consulting and Clinical Psychology, 85(11), 1080–1094. 10.1037/ccp0000245 29083223

[eat23831-bib-0029] Linardon, J. , Wade, T. , de la Piedad Garcia, X. , & Brennan, L. (2017b). Psychotherapy for bulimia nervosa on symptoms of depression: A meta‐analysis of randomized controlled trials. International Journal of Eating Disorders, 50, 1124–1136. 10.1002/eat.22763 28804915

[eat23831-bib-0030] Linardon, J. , Hindle, A. , & Brennan, L. (2017). Dropout from cognitive behavioral therapy for eating disorders: A meta‐analysis of randomized controlled trials. International Journal of Eating Disorders, 51, 381–391. 10.1002/eat.22850 29493805

[eat23831-bib-0031] Linardon, J. , Kothe, E. J. , & Fuller‐Tyskiewicz, M. (2019). Effectiveness of psychotherapy for bulimia nervosa and BED on self‐esteem improvement: Meta‐analysis. European Eating Disorders Review, 27, 109–123. 30623519 10.1002/erv.2662

[eat23831-bib-0032] Loucas, C. R. , Fairburn, C. G. , Whittington, C. , Pennant, M. E. , Stockton, S. , & Kendall, T. (2014). E‐therapy in the treatment and prevention of eating disorders: A systematic review and meta‐analysis. Behaviour Research and Therapy, 63, 122–131. 10.1016/j.brat.2014.09.011 25461787 PMC4271736

[eat23831-bib-0033] Miniati, M. , Callari, A. , Maglio, A. , & Calugi, S. (2018). Interpersonal psychotherapy for eating disorders: Current perspectives. Psychology Research and Behavior Management, 11, 353–369. 10.2147/PRBM.S120584 30233263 PMC6130260

[eat23831-bib-0034] Palavras, M. A. , Hay, P. , dos Santos Filho, C. A. , & Claudino, A. (2017). The effectiveness of psychological therapies in reducing weight and binge eating in people with bulimia nervosa and binge eating disorder who are overweight or obese—A critical synthesis and meta‐analyses. Nutrients, 9, 299. 10.3390/nu9030299 28304341 PMC5372962

[eat23831-bib-0035] Pittock, A. , Hodges, L. , & Lawrie, S. M. (2018). The effectiveness of internet‐delivered cognitive behavioral therapy for those with bulimic symptoms: A systematic review. BMC Research Notes, 11, 748. 10.1186/s13104-018-3843-2 30348226 PMC6196450

[eat23831-bib-0036] Pittock, A. , & Mair, E. (2010). Are psychotherapies effective in the treatment of anorexia nervosa? – A systematic review. Journal of the Indian Association of Children and Adolescent Mental Health, 6(3), 55–71.

[eat23831-bib-0037] Polnay, A. , James, V. A. W. , Hodges, J. L. , Murray, G. D. , Munro, C. , & Lawrie, S. M. (2014). Group therapy for people with bulimia nervosa: Systematic review and meta‐analysis. Psychological Medicine, 44, 2241–2254. 10.1017/S0033291713002791 24238470

[eat23831-bib-0038] Reas, D. L. , & Grilo, C. M. (2008). Review and meta‐analysis of pharmacotherapy for binge‐eating disorder. Obesity, 16, 2024–2038. 10.1038/oby.2008.333 19186327 PMC3650491

[eat23831-bib-0039] Solmi, M. , Wade, T. D. , Byrne, S. , Del Giovane, C. , Fairburn, C. G. , Ostinelli, E. G. , De Crescenzo, F. , Johnson, C. , Schmidt, U. , Treasure, J. , Favaro, A. , Zipfel, S. , & Cipriani, A. (2021). Comparative effectiveness and acceptability of psychological interventions for the treatment of adult outpatients with anorexia nervosa: A systematic review and network meta‐analysis. Lancet Psychiatry, 8, 215–224. 10.1016/S2215-0366(20)30566-6 33600749

[eat23831-bib-0040] Svaldi, J. , Schmitz, F. , Baur, J. , Hartmann, A. S. , Legenbauer, T. , Thaler, C. , von Wietersheim, J. , de Zwaan, M. , & Tuschen‐Caffier, B. (2019). Effectiveness of psychotherapies and pharmacotherapies for bulimia nervosa. Psychological Medicine, 49, 898–910. 10.1017/S0033291718003525 30514412

[eat23831-bib-0041] Thompson Brenner, H. , Glass, S. , & Westen, D. (2003). Implications for the treatment of bulimia nervosa: A meta‐analysis of effectiveness trials and a naturalistic study of treatment in the community. Clinical Psychology: Science and Practice, 10(3), 269–287. 10.1093/clipsy/bpg024

[eat23831-bib-0042] Van den Berg, E. , Houtzager, L. , de Vos, J. , Daemen, I. , Katsaragaki, G. , Kryotaki, E. , Cuijpers, P. , & Dekker, J. (2019). Psychological treatments for anorexia nervosa. European Eating Disorders Review, 27, 331–351. 10.1002/erv.2683 31124215

[eat23831-bib-0043] Vogel, E. N. , Singh, S. , & Accurso, E. C. (2021). A systematic review of cognitive behavior therapy and dialectical behavior therapy for adolescent eating disorders. Journal of Eating Disorders, 9(1), 1–38. 10.1186/s40337-021-00461-1 34663452 PMC8522082

[eat23831-bib-0044] Watson, H. J. , & Bulik, C. M. (2013). Update on the treatment of anorexia nervosa: Review of clinical trials, practice guidelines and emerging interventions. Psychological Medicine, 43, 2477–2500. 10.1017/S0033291712002620 23217606

[eat23831-bib-0045] Carter, F. A. , Jordan, J. , McIntosh, V. V. , Luty, S. E. , McKenzie, J. M. , Frampton, C. M. , Bulik, C. M. , & Joyce, P. R. (2011). The long‐term efficacy of three psychotherapies for anorexia nervosa: A randomized, controlled trial. International Journal of Eating Disorders, 44(7), 647–654. 21997429 10.1002/eat.20879

[eat23831-bib-0046] Cooper, Z. , & Fairburn, C. G. (2011). The evolution of “enhanced” cognitive behavior therapy for eating disorders: Learning from treatment nonresponse. Cognitive and Behavioral Practice, 18(3), 394–402. 23814455 10.1016/j.cbpra.2010.07.007 PMC3695554

[eat23831-bib-0047] Fairburn, C. G. (1981). A cognitive behavioural approach to the treatment of bulimia. Psychological Medicine, 11, 707–711. 6948316 10.1017/s0033291700041209

[eat23831-bib-0048] Fairburn, C. G. , & Beglin, S. J. (2008). Eating disorder examination questionnaire. Cognitive behavior therapy and eating disorders, 309, 313.

[eat23831-bib-0049] Fairburn, C. G. , Cooper, Z. , & Cooper, P. (1986). The clinical features and maintenance of bulimia nervosa. In K. D. Brownell & J. P. Foreyt (Eds.), Physiology, psychology and treatment of the eating disorders. Basic Books.

[eat23831-bib-0050] Fairburn, C. G. , Cooper, Z. , Doll, H. A. , O'Connor, M. E. , Palmer, R. L. , & Dalle Grave, R. (2013). Enhanced cognitive behaviour therapy for adults with anorexia nervosa: A UK–Italy study. Behaviour Research and Therapy, 51(1), R2–R8. 23084515 10.1016/j.brat.2012.09.010 PMC3662032

[eat23831-bib-0051] Fairburn, C. G. , Cooper, Z. , & Shafran, R. (2003). Cognitive behaviour therapy for eating disorders: A “transdiagnostic” theory and treatment. Behaviour Research and Therapy, 41(5), 509–528. 12711261 10.1016/s0005-7967(02)00088-8

[eat23831-bib-0052] Fairburn, C. G. , Cooper, Z. , Shafran, R. , Bohn, K. , Hawker, D. , Murphy, R. , & Straebler, S. (2008). Enhanced cognitive behavior therapy for eating disorders: The core protocol. In C. G. Fairburn (Ed.), Cognitive behavior therapy and eating disorders. Guilford Press.

[eat23831-bib-0053] Fairburn, C. G. , Marcus, M. D. , & Wilson, G. T. (1993). Cognitive behaviour therapy for binge eating and bulimia nervosa: A comprehensive treatment manual. In C. G. Fairburn & G. T. Wilson (Eds.), Binge eating: Nature, assessment, and treatment (pp. 361–404). Guilford Press.

[eat23831-bib-0054] Fordham, B. , Suganvam, T. , Edwards, K. , Hemming, K. , Howick, J. , Copsey, B. , Lee, H. , Kaidesoja, M. , Kirtley, S. , Hopewell, S. , Das Nair, R. , Howard, R. , Stallard, P. , Hamer‐Hunt, J. , Cooper, Z. , & Lamb, S. E. (2021a). Cognitive–behavioural therapy for a variety of conditions: An overview of systematic reviews and panoramic meta‐analysis. Health Technology Assessment, 25(9), 1–378. 10.3310/hta25090 PMC7957459 33629950

[eat23831-bib-0055] Fordham, B. , Suganavam, T. , Edwards, K. , Stallard, P. , Howard, R. , Das Nair, R. , Copsey, B. , Lee, H. , Howick, J. , & Hemming, K. (2021b). The evidence for cognitive behavioural therapy in any condition, population or context: A meta‐review of systematic reviews and panoramic meta‐analysis. Psychological Medicine, 51, 21–29. 10.1017/S0033291720005292 33455594 PMC7856415

[eat23831-bib-0056] Garner, D. M. (2004). Eating disorder inventory‐3 (EDI‐3). Professional manual. Psychological Assessment Resources.

[eat23831-bib-0057] Garner, D. M. , & Bemis, K. M. (1982). A cognitive‐behavioral approach to anorexia nervosa. Cognitive Therapy and Research, 6, 123–150.

[eat23831-bib-0058] Garner, D. M. , Olmstead, M. P. , & Polivy, J. (1983). Development and validation of a multidimensional eating disorder inventory for anorexia nervosa and bulimia. International Journal of Eating Disorders, 2(2), 15–34.

[eat23831-bib-0059] Hilbert, A. , Hoek, H. W. , & Schmidt, R. (2017). Evidence‐based clinical guidelines for eating disorders: International comparison. Current Opinion in Psychiatry, 30(6), 423–437. 28777107 10.1097/YCO.0000000000000360 PMC5690314

[eat23831-bib-0060] Kazdin, A. E. , Fitzsimmons‐Craft, E. E. , & Wilfley, D. E. (2017). Addressing critical gaps in the treatment of eating disorders. International Journal of Eating Disorders, 50(3), 170–189. 10.1002/eat.22670 28102908 PMC6169314

[eat23831-bib-0061] Khan, K. S. , Ter Riet, G. , Glanville, J. , Sowden, A. J. , & Kleijnen, J. (2001). Undertaking systematic reviews of research on effectiveness: CRD's guidance for carrying out or commissioning reviews (No 4 (2n)). Centre for Reviews and Dissemination.

[eat23831-bib-0062] Linardon, J. , de la Piedad Garcia, X. , & Brennan, L. (2017). Predictors, moderators, and mediators of treatment outcome following manualised cognitive‐behavioural therapy for eating disorders: A systematic review. European Eating Disorders Review, 25(1), 3–12. 27862611 10.1002/erv.2492

[eat23831-bib-0063] McIntosh, V. V. , Jordan, J. , Luty, S. E. , Carter, F. A. , McKenzie, J. M. , Bulik, C. M. , & Joyce, P. R. (2006). Specialist supportive clinical management for anorexia nervosa. International Journal of Eating Disorders, 39(8), 625–632. 16937382 10.1002/eat.20297

[eat23831-bib-0064] Mulkens, S. , & Waller, G. (2021). New developments in cognitive‐behavioural therapy for eating disorders (CBT‐ED). Current Opinion in Psychiatry, 34(6), 576–583. 34456306 10.1097/YCO.0000000000000745 PMC8500363

[eat23831-bib-0065] National Institute for Health and Care Excellence . (2017). *Eating disorders: recognition and treatment* [NICE guideline no. 69]. https://www.nice.org.uk/guidance/ng69 28654225

[eat23831-bib-0066] Roth, A. D. , & Pilling, S. (2007). The competencies required to deliver effective cognitive and behavioural therapy for people with depression and with anxiety disorders. Department of Health and Social Care.

[eat23831-bib-0067] Schmidt, U. , Magill, N. , Renwick, B. , Keyes, A. , Kenyon, M. , Dejong, H. , Lose, A. , Broadbent, H. , Loomes, R. , Yasin, H. , Watson, C. , Ghelani, S. , Bonin, E. M. , Serpell, L. , Richards, L. , Johnson‐Sabine, E. , Boughton, N. , Whitehead, L. , Beecham, J. , … Landau, S. (2015). The Maudsley outpatient study of treatments for anorexia nervosa and related conditions (MOSAIC): Comparison of the Maudsley model of anorexia nervosa treatment for adults (MANTRA) with specialist supportive clinical management (SSCM) in outpatients with broadly defined anorexia nervosa: A randomized controlled trial. Journal of Consulting and Clinical Psychology, 83(4), 796–807. 25984803 10.1037/ccp0000019

[eat23831-bib-0068] Shea, B. J. , Reeves, B. C. , Wells, G. , Thuku, M. , Hamel, C. , Moran, J. , Moher, D. , Tugwell, P. , Welch, V. , Kristjansson, E. , & Henry, D. A. (2017). AMSTAR 2: A critical appraisal tool for systematic reviews that include randomised or non‐randomised studies of healthcare interventions, or both. BMJ, 358, j4008. 10.1136/bmj.j4008 28935701 PMC5833365

[eat23831-bib-0069] Touyz, S. , le Grange, D. , Lacey, H. , Hay, P. , Smith, R. , Maguire, S. , Bamford, B. , Pike, K. M. , & Crosby, R. D. (2013). Treating severe and enduring anorexia nervosa: A randomized controlled trial. Psychological Medicine, 43(12), 2501–2511. 23642330 10.1017/S0033291713000949

[eat23831-bib-0070] Weissman, R. S. , Frank, G. K. W. , Klump, K. L. , Thomas, J. J. , Wade, T. , & Waller, G. (2017). The current status of cognitive behavioral therapy for eating disorders: Marking the 51st Annual Convention of the Association of Behavioral and Cognitive Therapies. International Journal of Eating Disorders, 50(12), 1444–1446. 10.1002/eat.22809 29210517

