# Supplementary material for: Cognitive behavioral therapy for eating disorders: A map of the systematic review evidence base
Source: Int J Eat Disord. 2022 Oct 31;56(2):295–313. doi: 10.1002/eat.23831 (PMC10092269; doi:10.1002/eat.23831)
Supplement: Supplementary file 1 — Appendix S1. Supporting information. [file EAT-56-295-s003.docx]

## S1. Excluded Papers with Reasons

**DARE criteria not fulfilled**

Attia, E. (2021). Anorexia nervosa treatment trials: time for new approaches. *The Lancet Psychiatry*, *8*(3), 170-171. https://doi.org/10.1016/S2215-0366(21)00038-9.

Bailey, A. P., Parker, A. G., Colautti, L. A., Hart, L. M., Liu, P., & Hetrick, S. E. (2014). Mapping the evidence for the prevention and treatment of eating disorders in young people. *Journal of eating disorders*, *2*(1), 1-12. <https://doi.org/10.1007/s40519-013-0003-5>

Beintner, I., Jacobi, C., & Taylor, C. B. (2012). Effects of an Internet‐based prevention program for eating disorders in the USA and Germany—A meta‐analytic review. *European Eating Disorders Review*, *20*(1), 1-8. <https://doi.org/10.1002/erv.1130>

Bohon, C. (2020). Evidence-based treatment of bulimia nervosa and binge-eating disorder. *Journal of the American Academy of Child and Adolescent Psychiatry,* *59*(10 Supplement), 133-134.

Citrome, L. (2019). Binge eating disorder revisited: What’s new, what’s different, what’s next. *CNS Spectrums*, *24*(S1), 4-13. <https://doi.org/10.1017/S1092852919001032>

Derenne, J. & Couturier, J. (2019). An overview of evidence-based treatments in child and adolescent eating disorders*. Journal of the American Academy of Child and Adolescent Psychiatry*, 58(10).

Fingeret, M. C., Teo, I., & Epner, D. E. (2014). Managing body image difficulties of adult cancer patients: lessons from available research. *Cancer*, *120*(5), 633-641. <https://doi.org/10.1002/cncr.28469>

Gorrell, S., Reilly, E. E., Brosof, L., & Le Grange, D. (2022). Use of Telehealth in the Management of Adolescent Eating Disorders: Patient Perspectives and Future Directions Suggested from the COVID-19 Pandemic. *Adolescent Health, Medicine and Therapeutics*, *13*, 45. 10.2147/AHMT.S334977

Herpertz, S., Hagenah, U., Vocks, S., von Wietersheim, J., Cuntz, U., & Zeeck, A. (2011). The diagnosis and treatment of eating disorders. *Deutsches Ärzteblatt International*, *108*(40), 678. https://doi.org/ 10.1016/S2215-0366(21)00038-9

Jassogne, C., & Zdanowicz, N. (2018). Management of adult patients with anorexia nervosa: a literature review. *Psychiatria Danub*, *30*(7), 533-6.

le Grange, D., & Schmidt, U. (2005). The treatment of adolescents with bulimia nervosa. *Journal of Mental Health*, *14*(6), 587-597.

Lock, J. (2015). An update on evidence-based psychosocial treatments for eating disorders in children and adolescents. *Journal of Clinical Child & Adolescent Psychology*, *44*(5), 707-721. <https://doi.org/10.1080/15374416.2014.971458>

Lock, J. (2020). Evidence-based treatment of anorexia nervosa. *Journal of the American Academy of Child and Adolescent Psychiatry,* *59*(10 Supplement), 133.

Lock, J., Kraemer, H. C., Jo, B., & Couturier, J. (2019). When meta-analyses get it wrong: response to treatment outcomes for anorexia nervosa: a systematic review and meta-analysis of randomized controlled trials. *Psychological Medicine*, *49*(4), 697-698. https://doi.org/10.1017/S003329171800329X

Lundgren, J. D., Danoff‐Burg, S., & Anderson, D. A. (2004). Cognitive‐behavioral therapy for bulimia nervosa: An empirical analysis of clinical significance. *International Journal of Eating Disorders*, *35*(3), 262-274. <https://doi.org/10.1002/eat.10254>

Mitchell, J. E., & Raymond, N. C. (1992). Cognitive-behavioral therapy in treatment of bulimia nervosa. *Psychobiology and treatment of anorexia nervosa and bulimia nervosa*, 307-327.

Mitchell, J.E., Roerig, J., & Steffen, K. (2012). An update on treatment strategies for bulimia nervosa. In D. Stein & Y. Latzer (Eds.), *Treatment and recovery of eating disorders.* Nova Science Publishers.

Pinto, T.F., Da Silva, F.G.C., De Bruin, V.M.S., De Bruin, & P.F.C. (2016). Night eating syndrome: How to treat it? *Revista da Associacao Medica Brasileira.* 62, 701-707.

Ramacciotti, C. E., Coli, E., Marazziti, D., Segura-García, C., Brambilla, F., Piccinni, A., & Dell’Osso, L. (2013). Therapeutic options for binge eating disorder. *Eating and Weight Disorders-Studies on Anorexia, Bulimia and Obesity*, *18*(1), 3-9.

Rutherford, L., & Couturier, J. (2007). A review of psychotherapeutic interventions for children and adolescents with eating disorders. *Journal of the Canadian Academy of Child and Adolescent Psychiatry*, *16*(4), 153.

Spattini, L., Rioli, G., Longo, F., Ferrari, S., & Galeazzi, G. M. (2017). An update on current clinical management of eating disorders. *Minerva Psichiatrica*, *58*(1), 54-69.

Wade, T. D. (2019). Recent research on bulimia nervosa. *Psychiatric Clinics*, *42*(1), 21-32. ttps://doi.org/10.1016/j.psc.2018.10.002.

Whitbread, J., & McGown, A. (1994). The treatment of bulimia nervosa: What is effective? A meta-analysis. *Indian Journal of Clinical Psychology*, *21*(2), 32–44.

### No CBT-only RCT data/comparison

Blanchet, C., Mathieu, M. È., St-Laurent, A., Fecteau, S., St-Amour, N., & Drapeau, V. (2018). A systematic review of physical activity interventions in individuals with binge eating disorders. Current Obesity Reports, 7(1), 76-88. https://doi.org/10.1007/s13679-018-0295-x

Cameron, L.C. (1999). Treatment of bulimia nervosa: A meta-analysis. The University of Mississippi.

Clery, P., Stahl, D., Ismail, K., Treasure, J., & Kan, C. (2017). Systematic review and meta‐analysis of the effectiveness of interventions for people with Type 1 diabetes mellitus and disordered eating. Diabetic medicine, 34(12), 1667-1675. https://doi.org/10.1111/dme.13509

Ciążyńska, J., & Maciaszek, J. (2022). Various Types of Virtual Reality-Based Therapy for Eating Disorders: A Systematic Review. *Journal of clinical medicine*, *11*(17), 4956. https://doi.org/10.3390/jcm11174956

Cuijpers, P., Donker, T., Weissman, M. M., Ravitz, P., & Cristea, I. A. (2016). Interpersonal psychotherapy for mental health problems: a comprehensive meta-analysis. American Journal of Psychiatry, 173(7), 680-687. https://doi.org/10.1176/appi.ajp.2015.15091141

Dölemeyer, R., Tietjen, A., Kersting, A., & Wagner, B. (2013). Internet-based interventions for eating disorders in adults: a systematic review. BMC Psychiatry, 13(1), 1-16. https://doi.org/10.1186/1471-244X-13-207

Fetahi, E., Søgaard, A. S., & Sjögren, M. (2022). Estimating the Effect of Motivational Interventions in Patients with Eating Disorders: A Systematic Review and Meta-Analysis. *Journal of personalized medicine*, *12*(4), 577. <https://doi.org/10.3390/jpm12040577>

Garvey, W. T., Mechanick, J. I., Brett, E. M., Garber, A. J., Hurley, D. L., Jastreboff, A. M., Nadolsky, K., Pessah-Pollack, R., & Plodkowski, R. (2016). American association of clinical endocrinologists and American college of endocrinology comprehensive clinical practice guidelines for medical care of patients with obesity. Endocrine Practice, 22, 1-203. https://doi.org/10.4158/EP161365.GL

Groff, S. E. (2015). Is enhanced cognitive behavioral therapy an effective intervention in eating disorders? A review. Journal of Evidence-Informed Social Work, 12(3), 272-288. https://doi.org/10.1080/15433714.2013.835756

Haas, V., Nadler, J., Crosby, R. D., Madden, S., Kohn, M., Le Grange, D., ... & Correll, C. U. (2022). Comparing randomized controlled trials of outpatient family‐based or inpatient multimodal treatment followed by outpatient care in youth with anorexia nervosa: Differences in populations, metrics, and outcomes. *European Eating Disorders Review*. https://doi.org/10.1002/erv.2907

Hagan, K. E., Christensen, K. A., & Forbush, K. T. (2020). A preliminary systematic review and meta-analysis of randomized-controlled trials of cognitive remediation therapy for anorexia nervosa. *Eating behaviors*, *37*, 101391. <https://doi.org/10.1016/j.eatbeh.2020.101391>

Heywood, S. E., Connaughton, J., Kinsella, R., Black, S., Bicchi, N., & Setchell, J. (2022). Physical Therapy and Mental Health: Scoping Review. *Physical Therapy*.

Hubbard, J. B. (2014). Psychotherapy outcome for eating disorders: A meta-analysis. Brigham Young University.

Lewandowski, L. M., Gebing, T. A., Anthony, J. L., & O'Brien, W. H. (1997). Meta-analysis of cognitive-behavioral treatment studies for bulimia. Clinical Psychology Review, 17(7), 703-718. https://doi.org/10.1016/S0272-7358(97)00026-3

Linardon, J., Fitzsimmons-Craft, E. E., Brennan, L., Barillaro, M., & Wilfley, D. E. (2019). Dropout from interpersonal psychotherapy for mental health disorders: A systematic review and meta-analysis. Psychotherapy Research, 29(7), 870-881. https://doi.org/10.1080/10503307.2018.1497215

Oustric, P., Gibbons, C., Beaulieu, K., Blundell, J., & Finlayson, G. (2018). Changes in food reward during weight management interventions–a systematic review. Obesity Reviews, 19(12), 1642-1658. https://doi.org/10.1111/obr.12754

Pilling, S., Fonagy, P., Allison, E., Barnett, P., Campbell, C., Constantinou, M., ... & Kendall, T. (2020). Long-term outcomes of psychological interventions on children and young people’s mental health: A systematic review and meta-analysis. PloS One, 15(11), e0236525. https://doi.org/10.1371/journal.pone.0236525

Ragnhildstveit, A., Slayton, M., Jackson, L. K., Brendle, M., Ahuja, S., Holle, W., ... & Robison, R. (2022). Ketamine as a Novel Psychopharmacotherapy for Eating Disorders: Evidence and Future Directions. *Brain Sciences*, *12*(3), 382. <https://doi.org/10.3390/brainsci12030382>

Reas, D. L., & Grilo, C. M. (2021). Psychotherapy and medications for eating disorders: Better together?. Clinical Therapeutics, 43(1), 17-39. https://doi.org/10.1016/j.clinthera.2020.10.006

Royal Australian and New Zealand College of Psychiatrists Clinical Practice Guidelines Team for Anorexia Nervosa. (2004). Australian and New Zealand clinical practice guidelines for the treatment of anorexia nervosa. Australian & New Zealand Journal of Psychiatry, 38(9), 659-670. ttps://doi.org/10.1177/0004867414555814

Rozakou-Soumalia, N., Dârvariu, Ş., & Sjögren, J. M. (2021). Dialectical behaviour therapy improves emotion dysregulation mainly in binge eating disorder and bulimia nervosa: a systematic review and meta-analysis. *Journal of personalized medicine*, *11*(9), 931. https://doi.org/10.3390/jpm11090931

Saure, E., Ålgars, M., Laasonen, M., & Raevuori, A. (2022). Cognitive Behavioral and Cognitive Remediation Strategies for Managing Co-Occurring Anorexia Nervosa and Elevated Autism Spectrum Traits. *Psychology research and behavior management*, *15*, 1005. 10.2147/PRBM.S246056

Scelles, C., & Bulnes, L. C. (2021). EMDR as treatment option for conditions other than PTSD: A systematic review. *Frontiers in psychology*, *12*. 10.3389/fpsyg.2021.644369

Schneider, J., Pegram, G., Gibson, B., Talamonti, D., Tinoco, A., Craddock, N., ... & Forshaw, M. (2022). A mixed‐studies systematic review of the experiences of body image, disordered eating, and eating disorders during the COVID‐19 pandemic. *International Journal of Eating Disorders*. <https://doi.org/10.1002/eat.23706>

Torous, J., Bucci, S., Bell, I. H., Kessing, L. V., Faurholt‐Jepsen, M., Whelan, P., ... & Firth, J. (2021). The growing field of digital psychiatry: current evidence and the future of apps, social media, chatbots, and virtual reality. *World Psychiatry*, *20*(3), 318-335. https://doi.org/10.1002/wps.20883

Torbahn, G., Brauchmann, J., Axon, E., Clare, K., Metzendorf, M. I., Wiegand, S., ... & Ells, L. J. (2022). Surgery for the treatment of obesity in children and adolescents. *Cochrane Database of Systematic Reviews*, (9).

Vancampfort, D., Vanderlinden, J., De Hert, M., Adamkova, M., Skjaerven, L. H., Catalan-Matamoros, D., ... & Probst, M. (2013). A systematic review on physical therapy interventions for patients with binge eating disorder. *Disability and rehabilitation*, *35*(26), 2191-2196.

https://doi.org/10.3109/09638288.2013.771707

Vocks, S., Tuschen‐Caffier, B., Pietrowsky, R., Rustenbach, S. J., Kersting, A., & Herpertz, S. (2010). Meta‐analysis of the effectiveness of psychological and pharmacological treatments for binge eating disorder. International Journal of Eating Disorders, 43(3), 205-217. <https://doi.org/10.1002/eat.20696>

Zinser, J., O’Donnell, N., Hale, L., & Jones, C. J. (2022). Multi‐family therapy for eating disorders across the lifespan: A systematic review and meta‐analysis. *European Eating Disorders Review*. <https://doi.org/10.1002/erv.2919>

### Non-English text

Abbate Daga, G., Quaranta, M., Notaro, G., Urani, C., Amianto, F., & Fassino, S. (2011). Family therapy and eating disorders in young female patients: State of the art. *Journal of Psychopathology*, *17*, 40-47.

Bacaltchuk, J., & Hay, P. (1999). Tratamento da bulimia nervosa: síntese das evidências. *Brazilian Journal of Psychiatry*, *21*, 184-187. https://doi.org/10.1590/S1516-44461999000300012

Dingemans, A. E., Bruna, M. J., & van Furth, E. F. (2001). Vreetbuistoornis: een overzicht. *Tijdschrift voor Psychiatrie*, *43*(5), 321-332. [Binge eating disorder: A review]

Duchesne, M., Appolinário, J. C., Rangé, B. P., Freitas, S., Papelbaum, M., & Coutinho, W. (2007). Evidências sobre a terapia cognitivo-comportamental no tratamento de obesos com transtorno da compulsão alimentar periódica. *Revista de Psiquiatria do Rio Grande do Sul*, *29*, 80-92. <https://doi.org/10.1590/S0101-81082007000100015>

Español-Armengol, N., & Miján-de-la-Torre, A. Topic 31 Nutrition in Behavioural Disorders Module 31.2 Nutrition in Bulimia.

Guzman, G. A. R., Lemus, C. A. D., Garcia, R. R., & Agraz, F. P. (2005). Cognitive behavioral therapy for binge eating disorder: A review. *Psiquiatria*, *21*(1).

Hilbert, A., & Braehler, E. (2012). Interpersonal psychotherapy for eating disorders: A systematic and practical review. *Verhaltenstherapie*, *22*, 149.

SBU. (2016) *[Treatment for Binge Eating Disorder]*. SBU report no 248: Stockholm: Swedish Agency for Health Technology Assessment and Assessment of Social Services.

Vancampfort, D., Vanderlinden, J., Pieters, G., De Herdt, A., Schueremans, A., Adriaens, A., ... & Probst, M. (2012). Het belang van bewegingsgerichte interventies in de multidisciplinaire behandeling van een eetbuistoornis: een literatuuronderzoek. *Tijdschrift voor Psychiatrie*, *54*(8), 719-730. [The importance of movement-directed interventions in the multidisciplinary treatment of binge eating disorder: An overview]

Vist, G. E., Reinar, L. M., Straumann, G. H., & Wisting, L. (2017). Treatment of Persons who Suffer from Both an Eating Disorder and Diabetes.

### Superceded

Galsworthy-Francis, L. (2012) *The development and exploration of the Experiences of Humiliation Scale (EHS) in an eating disordered population*: University of Leicester.

Hay, P.J., Bacaltchuk, J. (2000). Psychotherapy for bulimia nervosa and binging. *Cochrane Database Systematic Reviews*, 4. CD000562

Hay, P.J. & Bacaltchuk, J. (2001). Psychotherapy for bulimia nervosa and binging. *Cochrane Database Systematic Reviews*, 3. CD000562

Hay, P.J. & Bacaltchuk, J. (2003). Psychotherapy for bulimia nervosa and binging. *Cochrane Database Systematic Reviews,* 1. CD000562

Hay, P.J., & Bacaltchuk, J (2008). Bulimia nervosa. *BMJ Clinical Evidence*, 2008.

Hay, P.J., Bacaltchuk, J., & Stefano, S. (2004). Psychotherapy for bulimia nervosa and binging. *Cochrane Database Systematic Reviews,* 3**.** CD000562

Pratt, H.D. (2010). Psychotherapy in the age of pharmacology. *International Journal of Child and Adolescent Health,* 3, 137-42.

Pratt, H.D. (2012). Psychotherapy in the age of pharmacology.

Summerbell, C.D., Ashton, V., Campbell, K.J., Edmunds, L., Kelly, S., & Waters E. (2003) Interventions for treating obesity in children. *Cochrane Database Systematic Reviews,* 3. CD001872

### Too broad definition of CBT

Galsworthy-Francis, L., & Allan, S. (2014). Cognitive behavioural therapy for anorexia nervosa: A systematic review. *Clinical Psychology Review*, *34*(1), 54-72. <https://doi.org/10.1016/j.cpr.2013.11.001>

Hilbert, A., Petroff, D., Herpertz, S., Pietrowsky, R., Tuschen-Caffier, B., Vocks, S., & Schmidt, R. (2019). Meta-analysis of the effectiveness of psychological and medical treatments for binge-eating disorder. *Journal of Consulting and Clinical Psychology*, *87*(1), 91. https://doi.org/10.1037/ccp0000358

Hilbert, A., Petroff, D., Herpertz, S., Pietrowsky, R., Tuschen‐Caffier, B., Vocks, S., & Schmidt, R. (2020). Meta‐analysis on the long‐term effectiveness of psychological and medical treatments for binge‐eating disorder. *International Journal of Eating Disorders, 53*(9), 1353-1376. https://doi.org/10.1002/eat.23297

Le, L. K. D., Barendregt, J. J., Hay, P., & Mihalopoulos, C. (2017). Prevention of eating disorders: a systematic review and meta-analysis. *Clinical Psychology Review*, *53*, 46-58. <https://doi.org/10.1016/j.cpr.2017.02.001>

Spielmans, G. I., Benish, S. G., Marin, C., Bowman, W. M., Menster, M., & Wheeler, A. J. (2013). Specificity of psychological treatments for bulimia nervosa and binge eating disorder? A meta-analysis of direct comparisons. *Clinical Psychology Review*, *33*(3), 460-469. <https://doi.org/10.1016/j.cpr.2013.01.008>

Whittal, M. L., Agras, W. S., & Gould, R. A. (1999). Bulimia nervosa: A meta-analysis of psychosocial and pharmacological treatments. *Behavior Therapy*, *30*(1), 117-135. https://doi.org/10.1016/S0005-7894(99)80049-5

### Papers based on Berkman 2006 or Berkman 2015

Brownley, K. A., Berkman, N. D., Peat, C. M., Lohr, K. N., Cullen, K. E., Bann, C. M., & Bulik, C. M. (2016). Binge-eating disorder in adults: a systematic review and meta-analysis. *Annals of internal medicine*, *165*(6), 409-420. https://doi.org/10.7326/M15-2455

Brownley, K. A., Berkman, N. D., Sedway, J. A., Lohr, K. N., & Bulik, C. M. (2007). Binge eating disorder treatment: a systematic review of randomized controlled trials. *International Journal of Eating Disorders*, *40*(4), 337-348. <https://doi.org/10.1002/eat.20370>

Bulik, C. M., Berkman, N. D., Brownley, K. A., Sedway, J. A., & Lohr, K. N. (2007). Anorexia nervosa treatment: a systematic review of randomized controlled trials. *International Journal of Eating Disorders*, *40*(4), 310-320. <https://doi.org/10.1002/eat.20367>

Peat, C. M., Berkman, N. D., Lohr, K. N., Brownley, K. A., Bann, C. M., Cullen, K., Quattlebaum, M.J., & Bulik, C. M. (2017). Comparative effectiveness of treatments for binge‐eating disorder: Systematic review and network meta‐analysis. *European Eating Disorders Review*, *25*(5), 317-328. https://doi.org/10.1002/erv.2517

Shapiro, J. R., Berkman, N. D., Brownley, K. A., Sedway, J. A., Lohr, K. N., & Bulik, C. M. (2007). Bulimia nervosa treatment: a systematic review of randomized controlled trials. *International Journal of Eating Disorders*, *40*(4), 321-336. https://doi.org/10.1002/eat.20372

### Abstracts or erratum with no sufficient data for inclusion

Hay, P., Galletly, C., Carter, G., Andrews, G., Chinn, D., Forbes, D., ... & Ward, W. (2015). RANZCP Clinical practice guidelines for eating disorders. *Australian and New Zealand Journal of Psychiatry, 49*, 30-31. 1

Lock, J. (2016). Evidence-Based Psychosocial Treatments for Eating Disorders in Children and Adolescents. *Journal of the American Academy of Child & Adolescent Psychiatry*, *10*(55), 88-89.

Loucas, C., Pennant, M., Whittington, C., Naqvi, S., Sealey, C., Stockton, S., Kelvin, R., Fonagy, P., & Kendall, T. (2014). G130 E-therapies for mental health problems in children and young people: A systematic review and focus group investigation. *Archives of disease in childhood*, *99*(Suppl 1), 58-58. http://dx.doi.org/10.1136/archdischild-2014-306237.138

Stuhldreher, N., Konnopka, A., Wild, B., Herzog, W., Zipfel, S., Löwe, B., & König, H. H. (2012). Cost‐of‐illness studies and cost‐effectiveness analyses in eating disorders: A systematic review. *International Journal of Eating Disorders*, *45*(4), 476-491. https://doi.org/10.1002/eat.20977

### Not able to obtain full text

Cardi, V. & Treasure, J. (2010). Treatments in eating disorders: Towards future directions. *Minerva Psichiatrica*, 51, 191-206.

Mitchell, K. & Carr, A. (2000). Anorexia and bulimia. In Carr A (Ed). *What Works with Children, Adolescents and Adults? A Critical Review of Psychological Interventions with Children, Adolescents and their Families (pp-. 233-257).* Taylor & Frances/Routledge.

Pratt, H.D. (2017). Psychotherapy in the age of pharmacology (2nd edition). In: DE Greydanus JC, DR Patel, A Nazeer and J Merrick, editor. *Clincial aspects of psychopharmacology in childhood and adolescence (pp. 83-91).* Nova Science Publishers.

### Review of reviews

Butler, A. C., Chapman, J. E., Forman, E. M., & Beck, A. T. (2006). The empirical status of cognitive-behavioral therapy: a review of meta-analyses. *Clinical Psychology Review*, *26*(1), 17-31. https://doi.org/10.1016/j.cpr.2005.07.003

Costa, M. B., & Melnik, T. (2016). Effectiveness of psychosocial interventions in eating disorders: an overview of Cochrane systematic reviews. *Einstein (Sao Paulo)*, *14*, 235-277.

Jansingh, A., Danner, U. N., Hoek, H. W., & van Elburg, A. A. (2020). Developments in the psychological treatment of anorexia nervosa and their implications for daily practice. *Current Opinion in Psychiatry*, *33*(6), 534. <https://doi.org.10.1097/YCO.0000000000000642>.

Leichsenring, F., Steinert, C., Rabung, S., & Ioannidis, J. P. (2022). The efficacy of psychotherapies and pharmacotherapies for mental disorders in adults: an umbrella review and meta‐analytic evaluation of recent meta‐analyses. *World Psychiatry*, *21*(1), 133-145. https://doi.org/10.1002/wps.20941

### Only included a CBT1 vs CBT2 comparison

Farrand, P., & Woodford, J. (2013). Impact of support on the effectiveness of written cognitive behavioural self-help: a systematic review and meta-analysis of randomised controlled trials. Clinical Psychology Review, 33(1), 182-195. https://doi.org/10.1016/j.cpr.2012.11.001

Flodgren, G., Rachas, A., Farmer, A. J., Inzitari, M., & Shepperd, S. (2015). Interactive telemedicine: effects on professional practice and health care outcomes. Cochrane Database of Systematic Reviews, (9). <https://doi.org/10.1002/14651858.CD002098.pub2>

Greenwood, H., Krzyzaniak, N., Peiris, R., Clark, J., Scott, A. M., Cardona, M., ... & Glasziou, P. (2022). Telehealth Versus Face-to-face Psychotherapy for Less Common Mental Health Conditions: Systematic Review and Meta-analysis of Randomized Controlled Trials. *JMIR mental health*, *9*(3), e31780. doi:10.2196/31780

### Already included in the original search

Dahlenburg, S. C., Gleaves, D. H., & Hutchinson, A. D. (2019). Treatment outcome research of enhanced cognitive behaviour therapy for eating disorders: A systematic review with narrative and meta-analytic synthesis. *Eating Disorders*, *27*(5), 482-502. <https://doi.org/10.1080/10640266.2018.1560240>

Grenon, R., Carlucci, S., Brugnera, A., Schwartze, D., Hammond, N., Ivanova, I., McQuaid, N., Proulx, G., & Tasca, G. A. (2019). Psychotherapy for eating disorders: A meta-analysis of direct comparisons. *Psychotherapy Research*, *29*(7), 833-845. https://doi.org/10.1080/10503307.2018.1489162

Svaldi, J., Schmitz, F., Baur, J., Hartmann, A. S., Legenbauer, T., Thaler, C., von Wietersheim, J., de Zwaan, M., & Tuschen-Caffier, B. (2019). Effectiveness of psychotherapies and pharmacotherapies for bulimia nervosa. *Psychological Medicine*, *49*(6), 898-910. https://doi.org/10.1017/S0033291718003525

### Wrong population (bariatric)

David, L. A., Sijercic, I., & Cassin, S. E. (2020). Preoperative and post‐operative psychosocial interventions for bariatric surgery patients: a systematic review. *Obesity Reviews*, *21*(4), e12926. https://doi.org/10.1111/obr.12926

Newman, A. K. R., Herbozo, S., Russell, A., Eisele, H., Zasadzinski, L., Hassan, C., & Sanchez-Johnsen, L. (2021). Psychosocial interventions to reduce eating pathology in bariatric surgery patients: a systematic review. *Journal of Behavioral Medicine*, *44*(3), 421-436. <https://doi.org/10.1007/s10865-021-00201-5>

Sijercic, I., et al. (2020). "Preoperative and post-operative psychosocial interventions for bariatric surgery patients: A systematic review." Obesity Reviews 21(4): e12926.

### Wrong timepoint (prevention)

Hollis, C., Falconer, C. J., Martin, J. L., Whittington, C., Stockton, S., Glazebrook, C., & Davies, E. B. (2017). Annual Research Review: Digital health interventions for children and young people with mental health problems–a systematic and meta‐review. *Journal of Child Psychology and Psychiatry*, *58*(4), 474-503. <https://doi.org/10.1111/jcpp.12663>

Le, L. K. D., Tan, E. J., Perez, J. K., Chiotelis, O., Hay, P., Ananthapavan, J., ... & Mihalopoulos, C. (2022). Prevention of high body mass index and eating disorders: a systematic review and meta-analysis. *Eating and Weight Disorders-Studies on Anorexia, Bulimia and Obesity*, 1-15. https://doi.org/10.1007/s40519-022-01458-8

Levine, M. P. (2021). Prevention of eating disorders: 2020 in review. *Eating disorders*, *29*(2), 134-150. https://doi.org/10.1080/10640266.2021.1909794

Pellegrini, D., Grennan, L., Bhatnagar, N., McVey, G., & Couturier, J. (2022). Virtual prevention of eating disorders in children, adolescents, and emerging adults: a scoping review. *Journal of eating disorders*, *10*(1), 1-44. https://doi.org/10.1186/s40337-022-00616-8

Watson, H. J., Joyce, T., French, E., Willan, V., Kane, R. T., Tanner‐Smith, E. E., McCormack, J., Dawkins, J., Hoiles, K.J., & Egan, S. J. (2016). Prevention of eating disorders: A systematic review of randomized, controlled trials. *International Journal of Eating Disorders*, *49*(9), 833-862. <https://doi.org/10.1002/eat.22577>

### No control group in the synthesis

Linardon, J. (2018). Rates of abstinence following psychological or behavioral treatments for binge‐eating disorder: Meta‐analysis. *International Journal of Eating Disorders*, *51*(8), 785-797. https://doi.org/10.1002/eat.22897

Linardon, J., & Wade, T. D. (2018). How many individuals achieve symptom abstinence following psychological treatments for bulimia nervosa? A meta‐analytic review. *International Journal of Eating Disorders*, *51*(4), 287-294. <https://doi.org/10.1002/eat.22838>

Moberg, L. T., Solvang, B., Sæle, R. G., & Myrvang, A. D. (2021). Effects of cognitive-behavioral and psychodynamic-interpersonal treatments for eating disorders: a meta-analytic inquiry into the role of patient characteristics and change in eating disorder-specific and general psychopathology in remission. *Journal of Eating Disorders*, *9*(1), 1-12. https://doi.org/10.1186/s40337-021-00430-8

### Wrong outcome

Linardon, J., de la Piedad Garcia, X., & Brennan, L. (2017). Predictors, moderators, and mediators of treatment outcome following manualised cognitive‐behavioural therapy for eating disorders: A systematic review. *European Eating Disorders Review*, *25*(1), 3-12. <https://doi.org/10.1002/erv.249>

Vinchenzo, C., Lawrence, V., & McCombie, C. (2022). Patient perspectives on premature termination of eating disorder treatment: a systematic review and qualitative synthesis. *Journal of Eating Disorders*, *10*(1), 1-14. https://doi.org/10.1186/s40337-022-00568-z
