# Supplementary material for: Cognitive behavioral therapy for eating disorders: A map of the systematic review evidence base
Source: Int J Eat Disord. 2022 Oct 31;56(2):295–313. doi: 10.1002/eat.23831 (PMC10092269; doi:10.1002/eat.23831)
Supplement: Supplementary file 2 — Appendix S2. Supporting information. [file EAT-56-295-s002.docx]

## S2. Table 7a

*Summary Table of MAs Comparing CBT to Inactive Controls (ED-specific outcomes)*

| *CBT intensity* | | *Control* | *ED type* | *ED behaviors* | *ED psychopathology* | *Remission/abstinence* | *Weight/*  *BMI* |
| --- | --- | --- | --- | --- | --- | --- | --- |
| High |  | Inactive control | BED | **Y (2-11^X^ RCTs), Y (3-4 RCTs)** | **Y (4-9^X^ RCTs),** N (6 RCTs) | **Y (2-11^X^ RCTs), Y (4 RCTs)** | N (2-4^X^ RCTs), N (3 RCTs) |
|  |  |  | BN | **Y (1-9 RCTs)** | **Y (8 RCTs),** N (3-4 RCTs) | **Y (3-5 RCTs),** N (2 RCTs) | N (1 RCT) |
|  |  |  | Mixed | **Y (12 RCTs)** | **Y (4 RCTs)^X^,** N (4 RCTs)^X^ | **Y (8 RCTs)** | N (4 RCTs) |
|  | CBT-E |  | Mixed |  | **Y (3 RCTs)^X^** |  |  |
|  | CBT-BN |  | Mixed |  | **Y (7-8 RCTs),** N (9 RCTs)^X^ |  |  |
|  | “adapted” CBT-BN |  | BED | N (2 RCTs) |  |  |  |
|  |  |  | BN | **Y (5 RCTs)** | N (4 RCTs) | **Y (3 RCTs)** |  |
|  | group CBT |  | BED | **Y (10 RCTs)** | N ( 5 RCTs) | **Y (7 RCTs)** |  |
|  |  |  | BN | **Y (4 RCTs)** | N ( 2 RCTs) | **Y (2 RCTs)** |  |
| High and low | |  | BED |  | **Y (4-5 RCTs) ^X^,** N (5 RCTs) ^X^ |  |  |
|  |  |  | BN | **Y (5-7 RCTs)** | **Y (4 RCTs)** **^X^,** N (5 RCTs) ^X^ |  |  |
|  |  |  | Mixed | **Y (8 RCTs)^X^** | **Y (10-12 RCTs)^X^** |  |  |
| Low | |  | BED | **Y (1-19 RCTs)^X^, Y(7 RCTs),** N (1-2**^X^** RCTs**)** | **Y (6-13 ^X^ RCTs),** N (1 RCT)**^X^** | **Y (1-18 ^X^ RCTs), Y(6 RCTs)** | N (1-7 RCTs) |
|  |  |  | BN | **Y (1-2^X^ RCTs),** N (1-5 RCTs) | **Y (8 RCTs),** N (2-3 RCTs)^X^ | **Y (1-4 RCTs),** N (1-2**^X^** RCTs) | N (1 RCT) |
|  |  |  | AN | **Y (1 RCT)^X^,** N(1 RCT)**^X^** | **Y (1 RCT) ^X^,** N(1 RCT)**^X^** |  |  |
|  |  |  | EDNOS | **Y (1 RCT)** |  | N(1 RCT) | N (1 RCT) |
|  |  |  | Mixed | **Y (1-3 RCTs)** | **Y (6-8 RCTs)^X^** | **Y (4 RCTs),** N (1-3 RCTs) |  |

*Note*. The syntheses that excluded poor quality RCTs are underlined. The number of RCTs reported in parentheses refers to the number of RCTs in the synthesis/-es in question. The statistically significant results (Y) are bolded. Y= a statistically significant result in favour of CBT, N= no statistically significant differences. For a definition of “adapted”, please see Linardon 2017c. ^X^ = the result is from a low/critically low quality review

## Table 7b

*Summary Table of MAs Comparing CBT to Inactive Controls (non – ED-specific outcomes)*

| *CBT intensity* | | *Control* | *ED type* | *Depression* | *Other psychological* | *Quality of life* | *Dropouts* |
| --- | --- | --- | --- | --- | --- | --- | --- |
| High | | Inactive control | BED | **Y (4-9^X^ RCTs)** | N (1 RCT) |  | **Y (10 RCTs)^X^,** N (1 RCT) |
|  |  |  | BN | **Y (2 -6 RCTs)** | N (1 RCT) |  | N (9 RCTs) |
|  |  |  | AN |  |  |  | N (NR RCTs) |
|  |  |  | Mixed | **Y (7 RCTs)** | N (2 RCTs) |  | N (11 RCTs) |
|  | group CBT |  | BN | N(2**^X^** RCTs) |  |  |  |
| High and low | |  | BN | **Y (8 RCTs)** |  |  |  |
|  |  |  | Mixed |  |  | **Y (4 RCTs)** |  |
| Low | |  | BED | **Y (5-12^X^ RCTs)** | **Y (1 RCT)** |  | **Y (15 RCTs),** N (1 RCT) |
|  |  |  | BN | N (1-2 RCTs) | N (1-2 RCTs) |  | N (1 RCT) |
|  |  |  | EDNOS | N (1 RCT) | N (1 RCT) |  | N (1 RCT) |
|  |  |  | Mixed | N (1-3 RCTs) | **Y (1 RCT),** N (1-2 RCTs) |  | N (1-4 RCTs) |

*Note*. The syntheses that excluded poor quality RCTs are underlined. The number of RCTs reported in parentheses refers to the number of RCTs in the synthesis/-es in question. The statistically significant results (Y) are bolded. Y= a statistically significant result in favour of CBT, N= no statistically significant differences. ^X^ = the result is from a low/critically low quality review
