# Supplementary material for: Cognitive behavioral therapy for eating disorders: A map of the systematic review evidence base
Source: Int J Eat Disord. 2022 Oct 31;56(2):295–313. doi: 10.1002/eat.23831 (PMC10092269; doi:10.1002/eat.23831)
Supplement: Supplementary file 3 — Appendix S3. Supporting information. [file EAT-56-295-s001.docx]

## S3. Figure 2

## Changes Made in the Data Extraction Template as Compared to the CBT-O Research Project (Fordham et al., 2021a)

| **Items added** | **Items excluded** |
| --- | --- |
| Type of CBT (ED-focused or general CBT) | CBT overall no. of sessions, duration and frequency |
| Psychiatric comorbidity | Mechanism data |
| A separate data extraction template for MA data | Acceptability |
|  | Satisfaction |
|  | Economic analyses |
