# Supplementary material for: Cognitive behavioral therapy for eating disorders: A map of the systematic review evidence base
Source: Int J Eat Disord. 2022 Oct 31;56(2):295–313. doi: 10.1002/eat.23831 (PMC10092269; doi:10.1002/eat.23831)
Supplement: Supplementary file 4 — Appendix S4. Supporting information. [file EAT-56-295-s004.docx]

## S4. Details of the search strategy

**1: Original CBT-O search strategy example (MEDLINE)**

**MEDLINE**

**Database & platform:**

Ovid MEDLINE(R) Epub Ahead of Print, In-Process & Other Non-Indexed Citations, Ovid MEDLINE(R) Daily and Ovid MEDLINE(R) 1946 to Present

**Search filter:**

SIGN Systematic Review Search Filter for MEDLINE (OVID) (<http://www.sign.ac.uk/search-filters.html>)

**Date search conducted:**

**Original:** 25 April 2018 (for publication years 1992 to Present) [2967 references]

**Update:** 30 January 2019 (for publication years 2018 to 2019) [359 references]

1. (cognitive adj2 behavio?r adj3 (therap$ or theor$ or intervention$ or train$ or treatment$ or psychotherap$ or programme$ or program$ or method$ or approach$)).ti,ab,kw.

2. (cognitive adj2 behavio?ral adj3 (therap$ or theor$ or intervention$ or train$ or treatment$ or psychotherap$ or programme$ or program$ or method$ or approach$)).ti,ab,kw.

3. CBT.ti,ab,kw.

4. Cognitive Therapy/

5. or/1-4

6. Meta-Analysis as Topic/

7. meta analy$.tw.

8. metaanaly$.tw.

9. Meta-Analysis/

10. (systematic adj (review$1 or overview$1)).tw.

11. exp Review Literature as Topic/

12 or/6-11

13. cochrane.ab.

14. embase.ab.

15. (psychlit or psyclit).ab.

16. (psychinfo or psycinfo).ab.

17. (cinahl or cinhal).ab.

18. science citation index.ab.

19. bids.ab.

20. cancerlit.ab.

21. or/13-20

22. reference list$.ab.

23. bibliograph$.ab.

24. hand-search$.ab.

25. relevant journals.ab.

26. manual search$.ab.

27. or/22-26

28. selection criteria.ab.

29. data extraction.ab.

30. 28 or 29

31. Review/

32. 30 and 31

33. Comment/

34. Letter/

35. Editorial/

36. animal/

37. human/

38. 36 and 37

39. 36 not 38

40. or/33-35,39

41. 12 or 21 or 27 or 32

42. 41 not 40

43. 5 and 42

44. limit 43 to yr="1992-2018"

**2: Updated search strategy example (MEDLINE)**

**MEDLINE**

**Database & platform:**

Ovid MEDLINE(R) Epub Ahead of Print, In-Process & Other Non-Indexed Citations, Ovid MEDLINE(R) Daily and Ovid MEDLINE(R)

**Search filter:**

SIGN Systematic Review Search Filter for MEDLINE (OVID) (<http://www.sign.ac.uk/search-filters.html>)

**Date search conducted:**

**Original:** 25 April 2018 (for publication years 1992 to Present) [2967+359 (updated 30 January 2019) references]

**Eating disorder update:** 15 April 2021 (for publication years 2019 to Present)

1. (cognitive adj2 behavio?r adj3 (therap$ or theor$ or intervention$ or train$ or treatment$ or psychotherap$ or programme$ or program$ or method$ or approach$)).ti,ab,kw.

2. (cognitive adj2 behavio?ral adj3 (therap$ or theor$ or intervention$ or train$ or treatment$ or psychotherap$ or programme$ or program$ or method$ or approach$)).ti,ab,kw.

3. CBT.ti,ab,kw.

4. Cognitive Therapy/

5. or/1-4

6. Meta-Analysis as Topic/

7. meta analy$.tw.

8. metaanaly$.tw.

9. Meta-Analysis/

10. (systematic adj (review$1 or overview$1)).tw.

11. exp Review Literature as Topic/

12 or/6-11

13. cochrane.ab.

14. embase.ab.

15. (psychlit or psyclit).ab.

16. (psychinfo or psycinfo).ab.

17. (cinahl or cinhal).ab.

18. science citation index.ab.

19. bids.ab.

20. cancerlit.ab.

21. or/13-20

22. reference list$.ab.

23. bibliograph$.ab.

24. hand-search$.ab.

25. relevant journals.ab.

26. manual search$.ab.

27. or/22-26

28. selection criteria.ab.

29. data extraction.ab.

30. 28 or 29

31. Review/

32. 30 and 31

33. Comment/

34. Letter/

35. Editorial/

36. animal/

37. human/

38. 36 and 37

39. 36 not 38

40. or/33-35,39

41. 12 or 21 or 27 or 32

42. 41 not 40

43. 5 and 42

44. limit 43 to yr="2018-2021"

45. "eating disorder"[All Fields]

46. 44 and 45

**3: Updated search strategy example (MEDLINE)**

**MEDLINE**

**Database & platform:**

Ovid MEDLINE(R) Epub Ahead of Print, In-Process & Other Non-Indexed Citations, Ovid MEDLINE(R) Daily and Ovid MEDLINE(R)

**Search filter:**

SIGN Systematic Review Search Filter for MEDLINE (OVID) (<http://www.sign.ac.uk/search-filters.html>)

**Date search conducted:**

**Original:** 25 April 2018 (for publication years 1992 to Present) [2967+359 (updated 30 January 2019) references]

**Eating disorder update:** 12 September 2022 (for publication 15 June 2021 to Present)

1. (cognitive adj2 behavio?r adj3 (therap$ or theor$ or intervention$ or train$ or treatment$ or psychotherap$ or programme$ or program$ or method$ or approach$)).ti,ab,kw.

2. (cognitive adj2 behavio?ral adj3 (therap$ or theor$ or intervention$ or train$ or treatment$ or psychotherap$ or programme$ or program$ or method$ or approach$)).ti,ab,kw.

3. CBT.ti,ab,kw.

4. Cognitive Therapy/

5. or/1-4

6. Meta-Analysis as Topic/

7. meta analy$.tw.

8. metaanaly$.tw.

9. Meta-Analysis/

10. (systematic adj (review$1 or overview$1)).tw.

11. exp Review Literature as Topic/

12 or/6-11

13. cochrane.ab.

14. embase.ab.

15. (psychlit or psyclit).ab.

16. (psychinfo or psycinfo).ab.

17. (cinahl or cinhal).ab.

18. science citation index.ab.

19. bids.ab.

20. cancerlit.ab.

21. or/13-20

22. reference list$.ab.

23. bibliograph$.ab.

24. hand-search$.ab.

25. relevant journals.ab.

26. manual search$.ab.

27. or/22-26

28. selection criteria.ab.

29. data extraction.ab.

30. 28 or 29

31. Review/

32. 30 and 31

33. Comment/

34. Letter/

35. Editorial/

36. animal/

37. human/

38. 36 and 37

39. 36 not 38

40. or/33-35,39

41. 12 or 21 or 27 or 32

42. 41 not 40

43. 5 and 42

44. anorexia.tw

45. bulimia.tw

46. binge eating.tw

47. eating disorder.tw

48. 44 or 45 or 46 or 47

49. limit 48 to 15 June 2021 – 12 September 2022
